# Metabolic engineering of microbes for branched-chain biodiesel production with low-temperature property

**DOI:** 10.1186/s13068-015-0270-7

**Published:** 2015-06-24

**Authors:** Hui Tao, Daoyi Guo, Yuchen Zhang, Zixin Deng, Tiangang Liu

**Affiliations:** Key Laboratory of Combinatorial Biosynthesis and Drug Discovery, Ministry of Education and Wuhan University School of Pharmaceutical Sciences, Wuhan, 430071 China; Hubei Engineering Laboratory for Synthetic Microbiology, Wuhan Institute of Biotechnology, Wuhan, 430075 China; State Key Laboratory of Microbial Metabolism, School of Life Sciences and Biotechnology, Shanghai Jiao Tong University, 800 Dongchuan Road, Shanghai, 200240 China; Hubei Provincial Cooperative Innovation Center of Industrial Fermentation, Wuhan, 430068 China

**Keywords:** Biodiesel, Branched-chain esters, Metabolic engineering, Branched-chain amino acid biosynthesis, WS/DGAT, *E. coli*, *Pichia pastoris*

## Abstract

**Background:**

The steadily increasing demand for diesel fuels calls for renewable energy sources. This has attracted a growing amount of research to develop advanced, alternative biodiesel worldwide. Several major disadvantages of current biodiesels are the undesirable physical properties such as high viscosity and poor low-temperature operability. Therefore, there is an urgent need to develop novel and advanced biodiesels.

**Results:**

Inspired by the proven capability of wax ester synthase/acyl-coenzyme A, diacylglycerol acyltransferase (WS/DGAT) to generate fatty acid esters, de novo biosynthesis of fatty acid branched-chain esters (FABCEs) and branched fatty acid branched-chain esters (BFABCEs) was performed in engineered *Escherichia coli* through combination of the (branched) fatty acid biosynthetic pathway and the branched-chain amino acid biosynthetic pathway. Furthermore, by modifying the fatty acid pathway, we improved FABCE production to 273 mg/L and achieved a high proportion of FABCEs at 99.3 % of total fatty acid esters. In order to investigate the universality of this strategy, *Pichia pastoris* yeast was engineered and produced desirable levels of FABCEs for the first time with a good starting point of 169 mg/L.

**Conclusions:**

We propose new pathways of fatty acid ester biosynthesis and establish proof of concept through metabolic engineering of *E. coli* and *P. pastoris* yeast. We were able to produce advanced biodiesels with high proportions FABCEs and BFABCEs. Furthermore, this new strategy promises to achieve advanced biodiesels with beneficial low-temperature properties.

**Electronic supplementary material:**

The online version of this article (doi:10.1186/s13068-015-0270-7) contains supplementary material, which is available to authorized users.

## Background

With rapidly diminishing reserves of petroleum and increasing concerns about climate change, the development of renewable fuels as alternatives to fossil fuels has become a worldwide priority. Currently, the predominant alternative fuels for transportation are corn ethanol and soybean biodiesel, but they cannot satisfy the increasing demands [1–3]. Biodiesel, derived from transesterification of vegetable oils or animal fats with monohydric alcohols, is an environmentally attractive alternative for conventional diesel fuels [4, 5]. Compared with petroleum, biodiesel has a large number of advantages including inherent lubricity and low toxicity. However, large-scale commercial biodiesel production requires very large amounts of vegetable oil that will result in competition with food industries and agriculture [6, 7]. To circumvent these hurdles, several biosynthetic pathways have been engineered to produce advanced biofuels [8–12]. Since fatty acids are precursors that can be converted to various products, the fatty acid biosynthetic pathway is especially attractive among these pathways, and many strategies have been applied to increase fatty acid production, for example, overexpression of acetyl-CoA carboxylase and thioesterase [13–16], in vitro reconstitution assay [17], reversal of the β-oxidation cycle strategy [18], and modular optimization of multi-gene pathways [19].

However, fatty acids cannot be utilized directly as biofuel due to the ionic nature of their carboxyl groups [3]; many studies have focused on the direct biosynthesis of biodiesels. After the identification of a novel wax ester synthase/acyl-CoA, diacylglycerol acyltransferase (WS/DGAT), which can synthesize fatty acid esters from alcohols and fatty acyl-CoAs [20, 21], this process became more popular. In *Escherichia coli*, Steinbüchel’s group achieved 0.26 g/L fatty acid ethyl esters (FAEEs) with exogenous oleic acid [22], and Keasling’s group obtained 674 mg/L FAEEs without feeding fatty acids [6]. In addition, fed-batch cultivation and a dynamic sensor-regular system also improved FAEEs production to 0.922 and 1.5 g/L, respectively [23, 24]. Moreover, several groups also engineered *Saccharomyces cerevisiae* to produce FAEEs [25, 26], and Nielsen’s group achieved a FAEE production of 34 mg/L by chromosome engineering and achieved another 40 % increase in FAEE production by overexpression of two genes (*ACB1* and *gapN*) [27].

In general, biodiesels usually refer to fatty acid methyl esters (FAMEs) or FAEEs. However, many recent studies have reported enhanced ester biosynthesis, especially the work of Atsumi’s group, which demonstrated the biosynthesis of a multitude of esters by combination of various alcohols and a diversity of CoA molecules, achieving a high production of isobutyl acetate from glucose (17.2 g/L) [28]. In addition, some investigations focused on the production of medium-chain esters in *E. coli*, such as isobutyl acetate and isoamyl acetate as well as butyrate esters [29, 30]. In our previous work, we presented fatty acid short-chain ester (FASE) biosynthesis as an improvement of the low-temperature properties of traditional fuels. This was achieved by expression of WS/DGAT, which catalyzes the esterification of fatty acyl-CoAs and short-chain alcohols, synthesized from the 2-keto acid pathway by expression of 2-keto acid decarboxylase (ARO10) and an alcohol dehydrogenase (ADH2). After further engineering, the optimal strain produced 209 ± 2.6 mg/L FASEs with 50 % being fatty acid branched-chain esters (FABCEs) [31].

Although we have a chieved biosynthesis of FASEs, a higher proportion of FABCEs would still be desirable due to their improved low-temperature properties. One disadvantage of biodiesels is their poor performance at temperatures below 0 °C because crystals can plug fuel lines and filters, causing problems in fuel pumping and engine operation [32]. Structural features, such as chain length, degree of unsaturation, and branching of the chain, influence the physical and fuel properties of a fatty acid ester molecule [33]. As reported earlier, when branches were introduced into linear, long-chain esters, the intramolecular associations should be attenuated and the crystallization temperatures reduced; furthermore, methyl branches can improve the low-temperature properties of biodiesels without increasing their oxidative susceptibility [34]. As previously reported, FABCEs exhibit lower freezing point and have a lower cloud point. For example, the cloud points of isopropyl and 2-butyl soyate are −9 and −12 °C, while the cloud point of methyl soyate is 0 °C [32, 35].

**Fig. 1:**
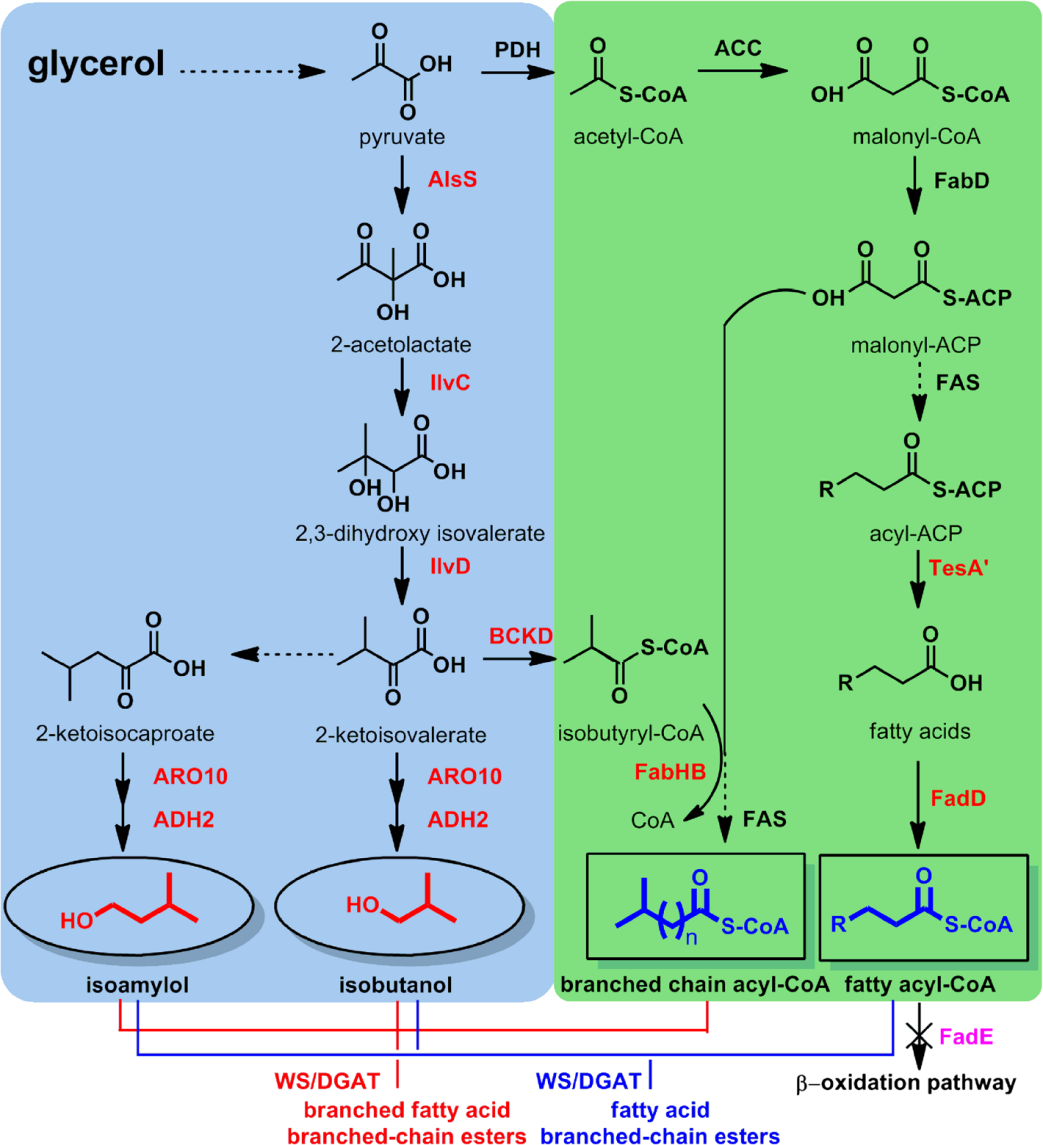
Engineered pathways for the production of FABCEs. Branched-chain alcohols (isobutanol and isoamylol) were produced through the branched-chain amino acid biosynthetic pathway by overexpression of *alsS*, *ilvC*, *ilvD*, *aro10*, and *adh2*. FABCEs were synthesized through esterification of branched-chain alcohols and fatty acyl-CoAs by overexpression of *ws/dgat*. Furthermore, BFABCEs were produced by overexpression of *ws/dgat* which can catalyze esterification of branched-chain alcohols and branched fatty acyl-CoAs produced by the overexpression of *bckd* and *fabHB*

Since both the fatty acid chain and the alcohol functionality contribute to the overall properties of a fatty acid ester molecule [33] and there are several studies which have reported the biosynthesis of branched-chain alcohols, such as isopropanol, isobutanol, and isoamylol, using branched-chain amino acid biosynthetic precursors in *E. coli* [36–39], here we present de novo biosynthesis of a higher proportion of FABCEs, including fatty acid isobutyl esters (FAIBEs) and fatty acid isoamyl esters (FAIAEs), which can be achieved through combination of the branched-chain amino acid biosynthetic pathways and fatty acid biosynthetic pathways in *E. coli* (Fig. 1). In addition, since some studies have reported the biosynthesis of branched-chain fatty acids [40–42], we successfully incorporated branched chains into both the fatty acid module and the alcohol module, yielding branched fatty acid branched-chain esters (BFABCEs) which expended the branched-chain ester variety. Finally, to test the universality of our strategy, we introduced the FABCE biosynthetic pathway into a eukaryote, *Pichia pastoris* yeast, resulting in a high production of FABCEs for the first time.

## Results

### De novo biosynthesis of FAIBEs and FAIAEs in *E. coli*

The valine and leucine biosynthetic pathways can generate two 2-keto acids: 2-ketoisovalerate and 2-ketoisocaproate. These can be converted into isobutanol and 3-methyl-1-butanol, respectively, via 2-keto-acid decarboxylase (KDC), and subsequently into alcohols by alcohol dehydrogenase (ADH) [36, 37]. We successfully generated FABCEs by combining fatty acid biosynthetic pathways with the valine and leucine biosynthetic pathways (Fig. 1). The *alsS* gene from *Bacillus subtilis* and *ilvCD* genes from *E. coli* were sub-cloned into the plasmid pDG102 [31], which expressed *ws/dgat*, *aro10*, *adh2*, and *fadD* genes under the control of T_7_ promoter. The resulting plasmid pDG104 was transformed into the *E. coli* BL21 (DE3) strain.

Recombinant *E. coli* cells were cultivated in a modified M9 medium and treated with isopropyl β-d-thiogalactopyranoside (IPTG) to induce expression of the genes mentioned above. Cell cultures were extracted 28 h after induction, and FABCEs quantified by gas chromatography–mass spectrometry (GC-MS, Additional file 1: Figure S1). Each FABCE was confirmed by corresponding standards that were synthesized by esterification of authentic fatty acid standards and alcohols (Additional file 2). The FABCE titer, yield, and productivity were 56 ± 6.3 mg/L, 2.8 ± 0.32 mg/g glycerol, and 2.0 ± 0.23 mg/L/h (Table 1), respectively. In this strain, 2015 ± 171.0 mg/L isobutanol and 838 ± 57.6 mg/L isoamylol were detected.

**Table 1:** Production of FABCEs in engineered *E. coli*

Strains	Concentration (mg/gDCW)	Yield (mg/g glycerol)	Productivity (mg/L/h)
BL21/pDG104	19 ± 1.3	2.8 ± 0.32	2.0 ± 0.23
BL21/pDG104/pDG105	142 ± 19.1	13.7 ± 2.05	9.8 ± 1.46
TL101/pDG104/pDG105	296 ± 26.8	8.9 ± 1.10	6.3 ± 0.79

Because we observed a large amount of branched-chain alcohols, we hypothesized that the amount of the other substrates, fatty acyl-CoAs, was not sufficient in our recombinant strain. Thus, increasing the fatty acyl-CoA supply should probably improve FABCE production. As previously described, the accumulation of fatty acyl-acyl carrier proteins (ACP) inhibits fatty acid biosynthesis [16, 43]. In order to overcome this problem, we attempted to overexpress a cytoplasmic thioesterase (TesA’ lacking the leader sequence for periplasmic secretion), which can alleviate the feedback inhibition of fatty acid biosynthesis by the accumulated fatty acyl-ACPs [13–16, 44, 45]. Therefore, we constructed the plasmid pDG105 expressing the *tesA*’ gene under the control of T_7_ promoter with a pSC101 replication origin. Coupled with pDG105, the modified strain (BL21/pDG104/pDG105) produced higher levels of FABCEs (273 ± 40.9 mg/L) with a high proportion of FABCEs at 99.3 % of total fatty acid ester production. FABCE yield and productivity were calculated to be 13.7 ± 2.05 mg/g glycerol and 9.8 ± 1.46 mg/L/h, respectively (Table 1). In addition, the branched-chain alcohols were quantified to be 513 ± 24.9 mg/L isobutanol and 499 ± 28.9 mg/L isoamylol. Thus, overexpression of TesA’ improved FABCE production by 4.9-fold compared to strain BL21/pDG104.

**Fig. 2:**
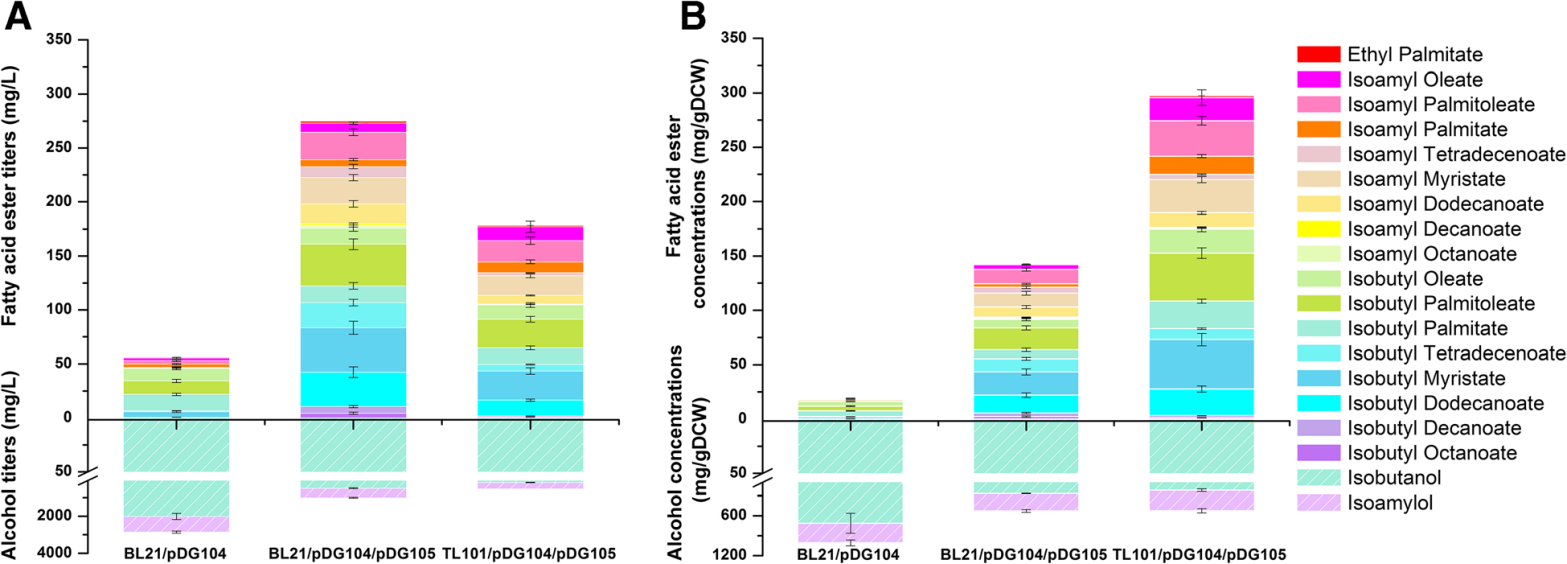
Production of FABCEs and branched-chain alcohols in engineered *E. coli* strains. The GC-MS quantification results showed the FABCE composition of engineered *E. coli* strains. Methyl pentadecanoic acid was used as the internal standard. Each FABCE was confirmed by corresponding standards that were synthesized by esterification of authentic fatty acid standards and alcohols (Additional file 2). All experiments were performed in triplicate and SD (standard deviation) is indicated. Each color indicates one particular product. **a** Titer of fatty acid esters and branched-chain alcohols in engineered *E. coli* strains. **b** Concentration of fatty acid esters and branched-chain alcohols in engineered *E. coli* strains when the cell mass was counted by OD_600_ with a coefficient of 0.3 gDCW/OD_600_

However, substantial accumulations of branched-chain alcohols were still present in cells, prompting us to take further measures to increase the fatty acyl-CoA supply. Since fatty acyl-CoAs can be degraded via the β-oxidation pathway, the plasmids pDG104 and pDG105 were transformed into *E. coli* TL101 which was constructed to block fatty acyl-CoA degradation by deleting the *fadE* operon in strain BL21 (DE3) as previously described [17]. Falling short to our expectations, the resulting strain (TL101/pDG104/pDG105) only achieved a titer of 177 ± 21.9 mg/L, which was even lower than that of strain BL21/pDG104/pDG105 (Fig. 2a). The yield and productivity of strain TL101/pDG104/pDG105 were 8.9 ± 1.10 mg/g glycerol and 6.3 ± 0.79 mg/L/h (Table 1). Similarly, 205 ± 15.9 mg/L isobutanol and 345 ± 36.2 mg/L isoamylol were measured. Nevertheless, in this study, the strain TL101/pDG104/pDG105 showed poor growth rate due to the large amount of intracellular FABCEs that may have adverse effects on the growth of cells (Additional file 3: Figure S2). When the cell mass was counted by optical cell densities at 600 nm (OD_600_) with a coefficient of 0.3 gDCW/OD_600_, the FABCE concentration of strain TL101/pDG104/pDG105 was calculated to be 296 ± 26.8 mg/gDCW (Table 1), which was almost twofold that of strain BL21/pDG104/pDG105 (Fig. 2b). Thus, blocking the β-oxidation pathway could also contribute to the FABCE production if inhibition of cell growth is reduced.

### Incorporation of branched chains into both fatty acid and alcohol modules for expanding ester biosynthesis in *E. coli*

Because we confirmed that engineered *E. coli* can produce FABCEs by coupling fatty acyl-CoAs with branched-chain alcohols, we further investigated whether branched chains could be incorporated into fatty acid carbon chains as well. As previously reported, *E. coli* cannot produce branched fatty acids (BFAs) naturally because the native *E. coli* β-ketoacyl-ACP synthase III (FabH) can only catalyze the condensation of malonyl-ACP and linear acetyl-CoA or propionyl-CoA in the first step of fatty acid elongation and the substrates required by FabH for production of branched chains are not present in *E. coli*, either. Fortunately, *B. subtilis* has a native pathway to supply branched starter substrates and FabH, which can accept branched starter substrates [46, 47].

**Fig. 3:**
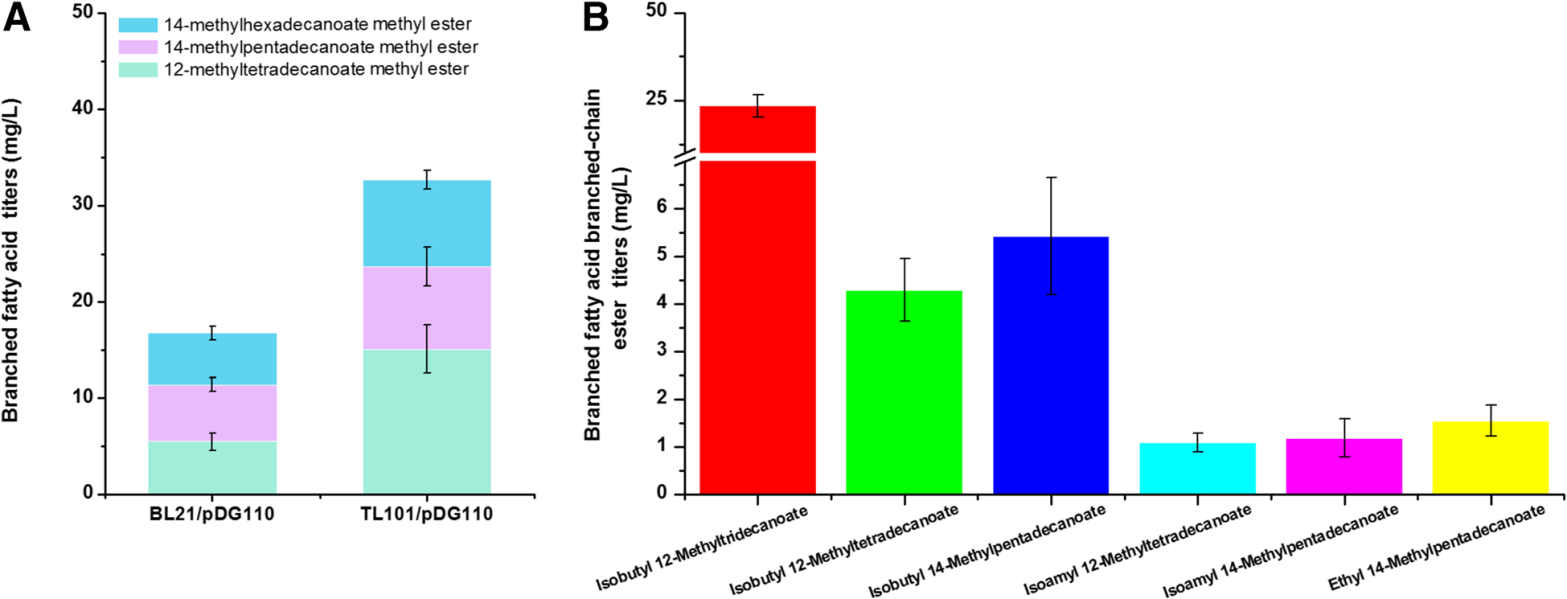
BFA and BFABCE titers in engineered *E. coli* strains. All experiments were performed in triplicate and SD is indicated. Each color indicated one particular product. **a** BFA titers in engineered *E. coli* strains. The total fatty acids were extracted and quantified by GC-MS after esterification with methanol. Pentadecanoic acid was used as the internal standard. 12-Methyltetradecanoate and 14-methylpentadecanoate were confirmed by their corresponding standard methyl esters which were made by esterification of BFA standards and methanol (Additional file 5). 14-Methylhexadecanoate had no available standard and was identified by comparing the mass spectrum to that of corresponding standard in the mass spectral libraries and that of relatively similar branched products. **b** BFABCE titers in engineered *E. coli* strains. The BFABCEs were quantified by GC-MS when methyl pentadecanoic acid was used as the internal standard. Except for isobutyl 12-methyltridecanoate, which was identified by comparing the mass spectrum to that of the corresponding standard in the mass spectral libraries, all BFABCEs were identified by the standard made by esterification of the corresponding fatty acid standards and alcohols (Additional file 5)

Consequently, in order to assemble a BFA biosynthetic pathway in *E. coli*, β-ketoacyl-ACP synthase III (FabHB) and branched-chain α-keto acid dehydrogenase complex (BCKD) from *B. subtilis* were overexpressed in *E. coli*. The resulting plasmid pDG110 was then transformed into BL21 (DE3) and TL101, respectively, and cultivated using the same methods as described above. BFAs were extracted by chloroform-methanol (1:1 by *v*/*v*) as described earlier [48] and quantitatively analyzed by GC-MS using pentadecanoic acid as an internal standard (Additional file 4: Figure S3A). The recombinant strains BL21/pDG110 and TL101/pDG110 produced BFAs namely 12-methyltetradecanoate, 14-methylpentadecanoate, and 14-methylhexadecanoate. 12-Methyltetradecanoate and 14-methylpentadecanoate were confirmed by their corresponding standard methyl esters, which were synthesized by esterification of BFA standards and methanol (Additional file 5). Besides, 14-methylhexadecanoate which has no available standard was identified by comparing the mass spectrum to that of corresponding standard in the mass spectral libraries and that of similar branched-chain products. The BFA production of strains BL21/pDG110 and TL101/ pDG110 were 17 ± 0.7 and 33 ± 5.4 mg/L, respectively (Fig. 3a). However, these results showed that although the deletion of the *fadE* gene increased BFA production by 94.1 %, the total BFA production was still very low compared to straight chain fatty acid production.

Because *E. coli* can produce BFAs using the strategy described above, we hypothesized that *E. coli* can produce BFABCEs by combining the BFA biosynthetic pathway and the branched-chain amino acid biosynthetic pathway. The plasmids pDG104, pDG105, and pDG110 were transformed into TL101 and cultivated and analyzed in the same way as previously described. The total ion chromatogram (TIC) of BFABCEs is shown in Additional file 4: Figure S3B, and the GC-MS quantification results of BFABCEs are shown in Fig. 3b. Except for isobutyl 12-methyltridecanoate, which was identified by comparing its mass spectrum to that of the corresponding standard in the mass spectral libraries, all BFABCEs were identified using the standard synthesized by the esterification of corresponding fatty acid and alcohols (Additional file 5). The recombinant strain TL101/pDG104/pDG105/pDG110 produced 35 ± 5.7 mg/L BFABCEs with a yield of 1.9 ± 0.28 mg/g glycerol and productivity of 1.3 ± 0.20 mg/L/h. This result indicated that we can incorporate a branched chain into both the fatty acid module and the alcohol module to produce new species of biodiesel with improved performance at low temperatures.

### Development of FABCE biosynthesis in *P. pastoris*

**Fig. 4:**
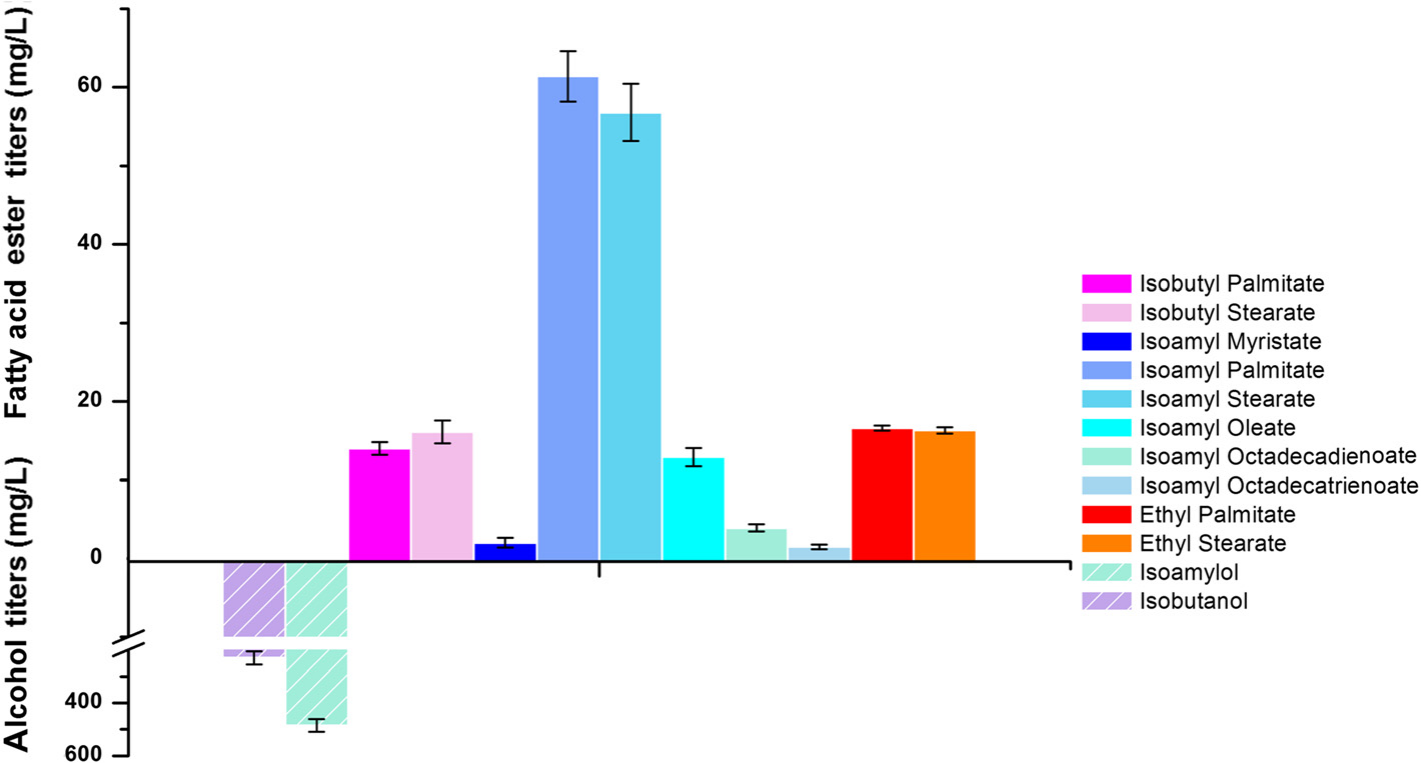
Titer of FABCEs and branched-chain alcohols in engineered *P. pastoris* yeast. The GC-MS quantification results show the FABCE composition of engineered *P. pastoris* yeast. Methyl pentadecanoic acid was used as the internal standard. Each FABCE was confirmed by corresponding standards synthesized by esterification of authentic fatty acid standards and alcohols (Additional file 2). All experiments were performed in triplicate, and SD is indicated. Each color indicates one particular product

Because the 2-keto acid biosynthetic pathway and the fatty acid biosynthetic pathway naturally exist in various microorganisms, we hypothesized that our FABCE biosynthetic strategy could be applied to other hosts. In fact, several groups have reported the biosynthesis of FAEEs from glucose by engineered *S. cerevisiae* [25–27, 49, 50]. *P. pastoris* yeast has been proven to be an excellent system to express various recombinant, heterologous proteins with a high cell density due to low hyper-glycosylation [51]. However, it has not been reported to produce FABCEs. Recently, *P. pastoris* yeast whole cell catalysts were constructed for biodiesel synthesis with intracellular expression of a lipase [52]. Therefore, we tested our hypothesis by developing a FABCE biosynthetic pathway in *P. pastoris*. First, we constructed a pDG10 vector for multiple gene expression under the control of GAP promoter. Three genes, *aro10*, *adh2*, and *ws/dgat*, were assembled into pDG10, yielding the plasmid pDG103. Subsequently, pDG103 was linearized and integrated into chromosome of *P. pastoris* GS115. Then, we cultured the recombinant strain *P. pastoris*/pDG103 in shake flasks and analyzed the products by GC-MS (Additional file 6: Figure S4). The recombinant strain produced 169 ± 11.7 mg/L of FABCEs (Fig. 4). Similarly, we detected 231 ± 25.4 mg/L of isobutanol and 485 ± 24.6 mg/L of isoamylol.

## Discussion

The commonly used biodiesels FAME and FAEE have many unwanted characteristics, especially poor low-temperature properties which can be affected by both fatty acid and alcohol moieties [33]. When methyl is replaced with a branched-chain group such as isobutyl and isoamyl, the resulting FABCEs display lower melting points [32, 35, 53, 54]. FABCEs may have higher energy densities compared with short or medium-chain esters. However, FABCE biosynthesis has not been reported yet. In this work, we provide strategies complementary to other engineering efforts. Specifically, we present de novo biosynthesis of high proportion of FABCEs, including FAIBEs and FAIAEs, by combining the branched-chain amino acid biosynthetic pathway and the fatty acid biosynthetic pathway. Because both fatty acid chain and alcohol portions can affect the low-temperature properties of biodiesels [33], branches were incorporated into fatty acid as well as alcohol chains, successfully generating BFABCEs. *E. coli* is an ideal host for genetic modification and metabolic engineering. Nevertheless, *E. coli* is a prokaryotic organism and has some disadvantages for industrial application, so we engineered *P. pastoris* yeast to produce FABCEs for the first time in order to test the universality of our strategy.

The engineered *E. coli* strain BL21/pDG104/pDG105 achieved a FABCE production of 273 ± 40.9 mg/L with a high proportion of FABCEs at 99.3 % of total fatty acid ester production (Fig. 2a). In other words, the engineered strain produced an extremely high proportion of FABCEs which can be utilized as advanced biodiesel with better low-temperature properties. The high proportion of FABCEs was most likely achieved due to several reasons. Steinbüchel’s group showed that the enzyme WS/DGAT accepts a broad range of various chain-length alcohols including short-chain alcohols such as ethanol and butanol. However, as reported in their work, the specificity of WS/DGAT toward C4–C8 alcohols was about five times that of ethanol [21]. This finding could explain why FABCEs were the predominant products in this work. Furthermore, besides the higher specificity of WS/DGAT toward C4–C8 alcohols, the overexpression of genes for branched-chain amino acid biosynthesis is another reason for the high proportion of FABCEs because these genes lead to the accumulation of a large amount of branched-chain alcohols such as isobutanol and isoamylol [2, 36–39] which are essential substrates for FABCE production.

On the other hand, when we introduced *fabHB* and *bckd* from *B. subtilis* into *E. coli*, the production of BFABCEs was only 22 % of all the fatty acid esters. This result can be explained by previous studies: overexpression of *fabHB* in *E. coli* results in two FabHs competing for malonyl-ACP [40, 42, 43]. However, the specific activity of *E. coli* FabH on acetyl-CoA is higher than the activity of *B. subtilis* FabHB to branched-chain substrates. Moreover, acetyl-CoA is native to *E. coli* in large amounts, whereas branched-chain substrates for FabHB are difficult to accumulate in *E. coli* [46, 55].

Because *E. coli* is not a robust host which is likely to suffer from phages, we presented this capability in *P. pastoris* yeast. The large amount (169 ± 11.7 mg/L) of FABCEs produced by *P. pastoris* yeast proved the universality of our strategy and gave us the implication that we can simply apply this strategy to other hosts that might be more potential for FABCE production. Compared with the highest FAEE production (50 mg/L, without precursor supplementation) achieved by *S. cerevisiae* [27], *P. pastoris* yeast described in this work indicates that *P. pastoris* yeast has greater potential to produce biodiesels because not only 32.9 mg/L FAEE but also 169 mg/L FABCEs were produced by *P. pastoris* yeast (Fig. 4). A potential explanation is that *P. pastoris* yeast shows superior expression of heterologous proteins [52]. The integration of genes in the chromosome and the use of the powerful GAP promoter also contributed to the high production of FABCEs by *P. pastoris* yeast. Because these strategies provide a more stable and efficient system, for example, in Nielsen’s work, the integration of genes results in a sixfold increase compared to the equivalent plasmid-based production in *S. cerevisiae* [27]. Above all, branched-chain alcohols with C4–C5 carbons are better substrates to WS/DGAT than ethanol [21, 56].

However, for both *E. coli* and *S. cerevisiae*, the titers are still below commercial levels, and further work will be necessary. Previous studies [57, 58], in which a specific residue was modified in WS/DGAT, show an increasing selectivity toward shorter chain fatty alcohol substrates, and this supports the potential for future efforts to improve the selectivity of WS/DGAT toward branched-chain alcohols by tailoring and modifying the enzyme WS/DGAT. Of course, studies focusing on finding more specific enzymes are also needed. The present work indicates that the bottleneck in FABCE production is the low proportion of fatty acyl-CoAs. However, this difficulty exists worldwide, and it might be alleviated by in vitro reconstitution [17] and modified enzymes used in the fatty acid biosynthesis pathway through rational protein design, as well as improved fatty acyl-CoAs supply. In addition, the universality of the present results indicates that other hosts might have greater potential for FABCE production, such as oleaginous yeast, which can accumulate a large amount of lipids [59].

## Conclusions

Several approaches to the low-temperature problems of biodiesels have been investigated including blending with conventional diesel fuel, winterization, and additives; this work provides a new strategy to solve the poor cold-flow performance of currently used biodiesels [33, 60]. The combination of the fatty acid biosynthetic pathway and the branched-chain amino acid biosynthetic pathway achieved a high proportion of FABCEs with improved low-temperature properties and high energy densities. The biosynthesis of FABCEs in both *E. coli* and *P. pastoris* proved the universality of our strategy although more work is needed to reach the level of commercialization. Nevertheless, using an approach that generates high proportions of FABCEs, the low-temperature performance of conventional biodiesels can be optimized. Our strategy can provide a start point for engineering more appropriate hosts to produce advanced biodiesels.

## Methods

### Materials

Restriction enzymes, Phusion polymerase, and Taq ligase were purchased from New England Biolabs (Beverly, MA, USA), and T5 exonuclease was purchased from Epicenter Biotechnologies (Madison, WI, USA). Plasmid miniprep kits, PCR purification kits, and gel extraction kits were purchased from Axygen (Alameda, CA, USA). All primers were synthesized by GENEWIZ (Suzhou, China). Fatty acid standards were purchased from TCI (Shanghai, China), and all the other reagents were purchased from Sigma-Aldrich (St. Louis, MO, USA).

### Plasmid and strain construction

Table 2 shows all plasmids used in this study. The sequences of all primers used in PCRs are listed in Table 3. The sequences of synthesized genes are listed in Additional file 7.

**Table 2:** Plasmids used in this study

Plasmids	Replication origin	Overexpressed genes	Resistance	Reference
pDG16	pBR322	P_T7_: *alsS*	Kan	This study
pDG17	pBR322	P_T7_: *ilvC*	Kan	This study
pDG18	pBR322	P_T7_: *ilvD*	Kan	This study
pDG19	pBR322	P_T7_: *ilvC* and *ilvD*	Kan	This study
pDG20	pBR322	P_T7_: *alsS*, *ilvC*, and *ilvD*	Kan	This study
pDG102	pBR322	P_T7_: *aro10*, *adh2*, *ws/dgat*, and *fadD*	Kan	Reference [31]
pDG104	pBR322	P_T7_: *ws/dgat*, *aro10*, *adh2*, *fadD*, *alsS*, *ilvC*, and *ilvD*	Kan	This study
pTL30	pBR322	P_T7_: *tesA*’	Kan	Reference [14]
pXC002	pSC101	P_T7_	Amp	Not published
pDG105	pSC101	P_T7_: *tesA*’	Amp	This study
pDG25	pBBR1	P_T7_: *bckd*	Cam	This study
pDG110	pBBR1	P_T7_: *fabHB* and *bckd*	Cam	This study
pDG10	pBR322	P_GAP_: *his4*	G418 and His	This study
pDG11	pBR322	P_GAP_: *ws/dgat*	G418	This study
pDG12	pBR322	P_GAP_: *aro10*	G418	This study
pDG13	pBR322	P_GAP_: *adh2*	G418	This study
pDG14	pBR322	P_GAP_: *ws/dgat*	G418 and His	This study
pDG15	pBR322	P_GAP_: *ws/dgat* and *aro10*	G418 and His	This study
pDG103	pBR322	P_GAP_: *ws/dgat*, *aro10*, and *adh2*	G418 and His	This study

**Table 3:** Primers used for PCR amplification of genes

Primer name	Primer sequence (5′-3′)
ilvC-XbaI	GTATCTAGAAAGAGGAGATATAATGGCTAACTACTTCAATACACTGAATCTG
ilvC-SpeI-BamHI	ACTGGATCCACTAGTTTAACCCGCAACAGCAATACGTT
ilvD-XbaI	GTATCTAGAAAGAGGAGATATAATGCCTAAGTACCGTTCCGCCA
ilvD-SpeI-BamHI	ACTGGATCCACTAGTTTAACCCCCCAGTTTCGATTTATCG
BCKD-SacI	AGATGAGCTCATGGCAACTGAGTATGACGTAGTCATTC
BCKD-SpeI	AGTACTAGTTTAGTAAACAGATGTCTTCTCGTCAATCG
fabHB-BamHI	ACTGGATCCAAGAAGGAGATATAATGTCAAAAGCAAAAATTACAGCTATCGG
fabHB-XhoI	TGTCTCGAGTTACATCCCCCATTTAATAAGCAATCCTG
WS/DGAT-SfuI	AGGTTTCGAAATGAGACCATTACACCCTATTGATTTC
WS/DGAT-EcoRI	GACGAATTCTTAGTTGGCAGTCTTAATGTCTTCTTG
HIS4-BamHI	AATGGATCCCTCGCAGCTGATGAATATCTTGTG
HIS4-BglII	CAAGGATCCACCGATACCAGGATCTTGCCAT
ARO10-SfuI	GCGTTCGAAATGGCACCTGTTACAATTGAAAAGTTC
ARO10-EcoRI	CCGTCTAGACTATTTTTTATTTCTTTTAAGTGCCGCTG
ADH2-SfuI	CCGTTCGAAATGTCTATTCCAGAAACTCAAAAAGCC
ADH2-EcoRI	GCGGAATTCTTATTTAGAAGTGTCAACAACGTATCTACCAG

### Plasmids for FAIBE and FAIAE production

The *alsS* gene [GenBank: 936852] from *B. subtilis* was synthesized using GenScript (Nanjing, China) with the sequence of the ribosome binding site (rbs) and cloned into the XbaI-BamHI sites of the plasmid pET28a (+), yielding pDG16. The *ilvC* [GenBank: 948286] and *ilvD* [GenBank: 948277] genes were amplified from *E. coli* genomic DNA with primers ilvC-XbaI/ilvC-SpeI-BamHI and ilvD-XbaI/ilvD-SpeI-BamHI, and the PCR products were cloned into pET28a (+), yielding pDG17 and pDG18, respectively. The XbaI-SalI fragment from pDG17 was cloned into pDG18 yielding pDG19. Subsequently, the XbaI-SalI fragment of pDG19 was cloned into pDG16 yielding pDG20. The XbaI-SalI fragment of pDG20 was cloned into the NheI-XhoI sites of pDG102 [31], yielding pDG104. The truncated *tesA*’ gene [GenBank: 945127] from pTL30 [14] was cloned under the control of T_7_ promoter of pXC002 with a pSC101 replication origin, yielding pDG105.

### Plasmids for BFA production

The *bckd* [GenBank: CP010052] and *fabHB* [GenBank: 939306] genes were amplified from *B. subtilis* genomic DNA with the primers fabHB-BamHI/fabHB-XhoI and BCKD-SacI/BCKD-SpeI. The *bckd* fragment was inserted into the SacI-SpeI sites of pBBR1MCS1, yielding pDG25. The PCR fragment of the *fabHB* gene was digested by SacI-SpeI and inserted into the BamHI-XhoI sites of pDG25, yielding pDG110.

### Plasmids for FABCE production in *P. pastoris*

First, a constitutive expression vector that can express multiple genes under the control of GAP promoter in *P. pastoris* was constructed as follows: The *his4* gene [GenBank: 850327] was amplified from the plasmid pPIC3.5k with the primers HIS4-BamHI and HIS4-BglII. The PCR fragment was inserted into the BamHI site of pGAPZα A, yielding pDG10, which can constitutively overexpress multiple genes in *P. pastoris*. Then, the synthesized *ws/dgat* gene [GenBank: 5984342] was amplified with the primers WS/DGAT-SfuI/WS/DGAT-EcoRI and cloned into the SfuI-EcoRI sites of pGAPZα A, yielding pDG11. The *aro10* [GenBank: 851987] and *adh2* [GenBank: 855349] genes from *S. cerevisiae* YPH499, coding 2-keto acid decarboxylase and alcohol dehydrogenase, were amplified from genomic DNA with the primers ARO10-SfuI/ARO10-EcoRI and ADH2-SfuI/ADH2-EcoRI and cloned into the SfuI-EcoRI sites of pGAPZα A, yielding pDG12 and pDG13, respectively. The BamHI-BglII fragment from pDG11 was inserted into the BamHI site of pDG10, resulting in the plasmid pDG14. Similarly, the BamHI-BglII fragment from pDG12 was inserted into the BamHI site of pDG14, yielding pDG15. A similar strategy was used to clone the *adh2* gene into pDG15, yielding pDG103.

### Shake-flask cultures

Table 4 shows all the strains used in this study. For *E. coli*, the corresponding plasmids were transformed into BL21 (DE3) or TL101 competent cells with suitable antibiotics, respectively, and several single colonies were pre-cultivated in a 10 mL LB medium in 50-mL flasks with proper antibiotics at 30 °C for 12 h. Cells were collected and resuspended with 100 mL modified M9 medium as described previously [31] in 500-mL flasks and shaken in a rotary shaker at 220 rpm and 30 °C. When OD_600_ reached 0.8, IPTG was added to a final concentration of 0.1 mM. Cells were harvested 28 h after induction. All experiments were performed in triplicate. For *P. pastoris*, pDG103 was linearized using SacI and transformed into competent cells of *P. pastoris* GS115 via electroporation. The positive transformants harboring chromosomal integration of *ws/dgat*, *adh2*, and *aro10* expression cassettes were preliminarily selected on SC-His plates supplemented with G418. Single colonies of harboring expression cassettes were used to inoculate 10 ml YPD medium in 50-mL flasks at 30 °C for 12 h. Cells were collected and resuspended with 100 mL YPD medium in 500-mL flasks and shaken in a rotary shaker at 220 rpm and 30 °C. Cells were harvested after 28 h. All experiments were performed in triplicate.

**Table 4:** Strains used in this study

Strains	Description	Reference
BL21(DE3)	*E. coli B dcm ompT hsdS(r*_*B*_ *ˉm*_*B*_ *ˉ)gal*	Invitrogen
TL101	*E. coli* BL21(DE3): Δ*fadE*	Reference [17]
BL21/pDG104	BL21(DE3) derivative;{pDG104}	This study
BL21/pDG104/pDG105	BL21(DE3) derivative;{pDG104, pDG105}	This study
TL101/pDG104/pDG105	BL21(Δ*fadE*) derivative;{pDG104, pDG105}	This study
BL21/pDG110	BL21(DE3) derivative;{pDG110}	This study
TL101/pDG110	BL21(Δ*fadE*) derivative;{pDG110}	This study
TL101/pDG104/pDG105/pDG110	BL21(Δ*fadE*) derivative;{pDG104, pDG105, pDG110}	This study
GS115	*his4*, *Mut* ^*+*^	Biovector
*P. pastoris*/pDG103	GS115 derivative;{pDG103}	This study

### Analysis methods

#### GC-MS analysis of fatty acid esters and branched-chain alcohols

Fatty acid esters were extracted using a previously published method [31]. GC-MS analysis of fatty acid esters dissolved in hexane phase was performed and quantified using a TRACE Ultra gas chromatograph connected to a TSQ Quantum XLS triple quadrupole mass spectrometer (GC-MS, Thermo Scientific Inc., San Jose, CA, USA). The hexane phase was analyzed on a TR-WAXMS capillary column (30 m × 0.25 mm × 0.25 μm), following a 1-μL injection with an auto-sampler (AI/AS 3000). Injections were performed in split mode with a split ratio of 20:1. Helium was used as the carrier gas with a flow rate of 1 mL/min. The inject inlet and ion source temperatures were 220 and 240 °C, respectively. The temperature sequence was programmed as follows: 100 °C as an initial temperature for 5 min, then a 6 °C/min ramp to 240 °C, held at 240 °C for 10 min. For identification of fatty acid esters, each fatty acid ester standard was synthesized by esterification of individual fatty acids with corresponding alcohols. For quantification, methyl pentadecanoic acid was used as an internal standard in all samples.

The branched-chain alcohol content of the shake-flask samples was extracted by hexane and analyzed by GC-MS as described previously [31]. Authentic standards were used to quantify isobutanol and isoamylol.

## Additional files

Additional file 1: Figure S1. GC-MS TIC of FABCEs in *E. coli* strains. All experiments were performed in triplicate. Identified substances: (1) isobutyl octanoate; (2) isoamyl octanoate; (3) isobutyl decanoate; (4) isoamyl decanoate; (5) isobutyl dodecanoate; (6) isoamyl dodecanoate; (7) isobutyl myristate; (8) isobutyl tetradecenoate; (9) ethyl palmitate; (10) isoamyl myristate; (11) isoamyl tetradecenoate; (12) isobutyl palmitate; (13) isobutyl palmitoleate; (14) (15) isoamyl palmitate; (16) isoamyl palmitoleate; (17) isobutyl oleate; (18) isoamyl oleate; (☆) 2-phenylethanol; (IS) methyl pentadecanoic acid (internal standard). Additional file 2:
**Synthetic FABCE standards.** The FABCE standards were synthesized by esterification of authentic fatty acid standards and corresponding branched-chain alcohols using approaches published in reference [48]. Additional file 3: Figure S2. OD_600_ curves of engineered *E. coli* strains in shake flasks. All experiments were performed in triplicate, and SD is indicated. Additional file 4: Figure S3. GC-MS TIC of BFAs and BFABCEs in *E. coli* strains. All experiments were performed in triplicate. (A) GC-MS TIC of BFAs. Identified substances: (1) 12-methyltetradecanoate methyl ester; (2) 14-methylpentadecanoate methyl ester; (3) 14-methylhexadecanoate methyl ester; (IS) methyl pentadecanoic acid (internal standard). (B) GC-MS TIC of BFABCEs. Identified substances: (1) isobutyl 12-methyltridecanoate; (2) ethyl 14-methylpentadecanoate; (3) isobutyl 12-methyltetradecanoate; (4) isobutyl 14-methylpentadecanoate; (5) isoamyl 12-methyltetradecanoate; (6) isoamyl 14-methylpentadecanoate; (IS) methyl pentadecanoic acid (internal standard). Additional file 5:
**Synthetic branched fatty acid ester standards.** The branched fatty acid ester standards were synthesized by esterification of authentic branched fatty acid standards and corresponding alcohols using approaches in detailed in reference [48]. Additional file 6: Figure S4. GC-MS TIC of FABCEs in *P. pastoris* yeast. *P. pastoris* GS115 is the negative control. All experiments were performed in triplicate. Identified substances: (1) ethyl palmitate; (2) isoamyl myristate; (3) isobutyl palmitate; (4) ethyl stearate; (5) isoamyl palmitate; (6) isobutyl stearate; (7) isoamyl stearate; (8) isoamyl oleate; (9) isoamyl octadecadienoate; (10) isoamyl octadecatrienoate; (IS) methyl pentadecanoic acid (internal standard). Additional file 7:
**The sequences of synthesized** ***ws/dgat*** **and** ***alsS*** **gene.**

## Abbreviations

ACP: Acyl carrier protein
BFAs: Branched fatty acids
BCKD: Branched-chain α-keto acid dehydrogenase complex
BFABCEs: Branched fatty acid branched-chain esters
CoA: Coenzyme A
DCW: Dry cell weight
FABCEs: Fatty acid branched-chain esters
FabH: β-ketoacyl-ACP synthase III
FAEEs: Fatty acid ethyl esters
FAIAEs: Fatty acid isoamyl esters
FAIBEs: Fatty acid isobutyl esters
FAME: Fatty acid methyl ester
FASEs: Fatty acid short-chain esters
GC-MS: Gas chromatography–mass spectrometry
IPTG: Isopropyl β-d-thiogalactopyranoside
LB: Luria-Bertani
rbs: Ribosome binding site
OD_600_: Optical cell densities at 600 nm
TIC: Total ion chromatogram
WS/DGAT: Wax ester synthase
YPD: Yeast extract peptone dextrose medium
