# Supplementary figures and images for: Metabolic engineering of microbes for branched-chain biodiesel production with low-temperature property
Source: Biotechnol Biofuels. 2015 Jun 24;8:92. doi: 10.1186/s13068-015-0270-7 (PMC4483204; doi:10.1186/s13068-015-0270-7)

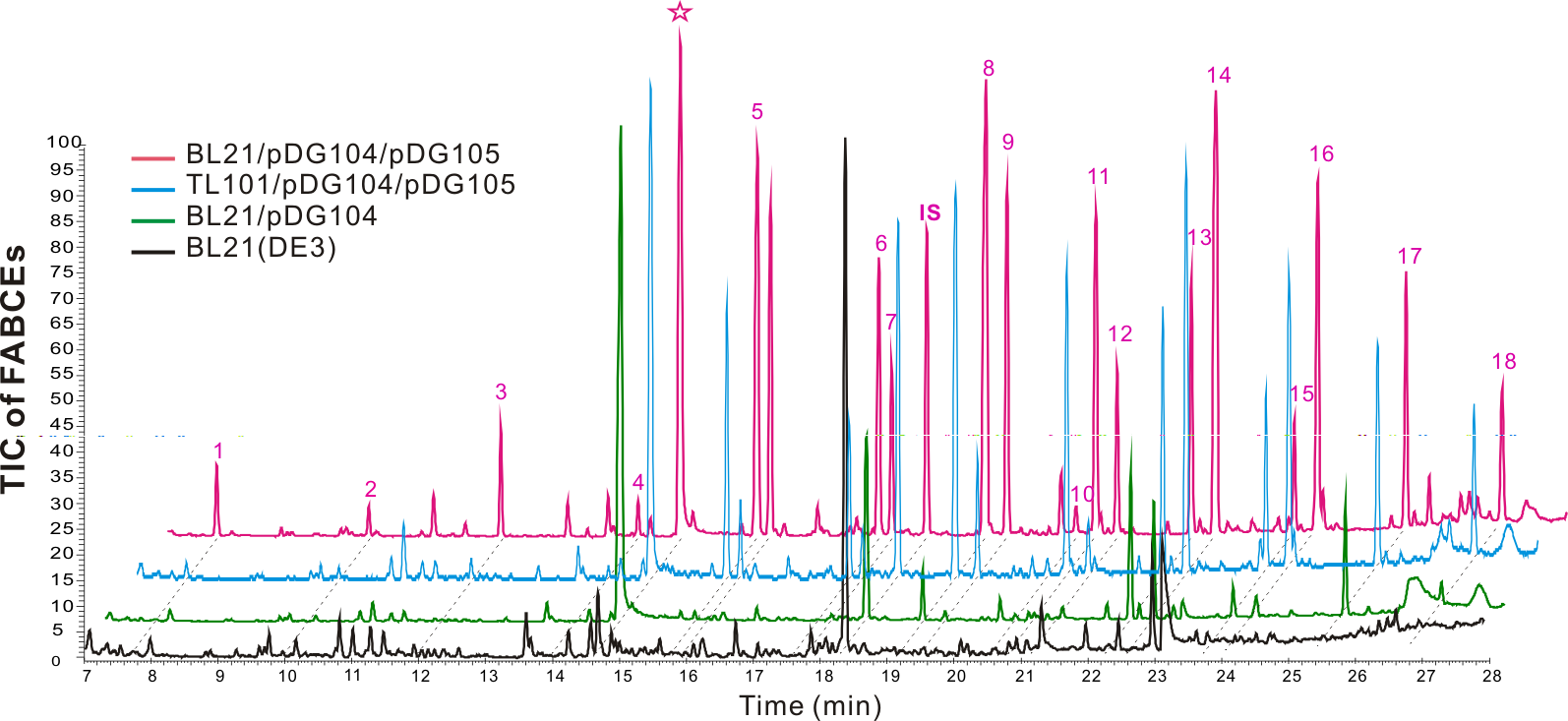

Supplement: Additional file 1: Figure S1. — GC-MS TIC of FABCEs in E. coli strains. All experiments were performed in triplicate. Identified substances: (1) isobutyl octanoate; (2) isoamyl octanoate; (3) isobutyl decanoate; (4) isoamyl decanoate; (5) isobutyl dodecanoate; (6) isoamyl dodecanoate; (7) isobutyl myristate; (8) isobutyl tetradecenoate; (9) ethyl palmitate; (10) isoamyl myristate; (11) isoamyl tetradecenoate; (12) isobutyl palmitate; (13) isobutyl palmitoleate; (14) (15) isoamyl palmitate; (16) isoamyl palmitoleate; (17) isobutyl oleate; (18) isoamyl oleate; (☆) 2-phenylethanol; (IS) methyl pentadecanoic acid (internal standard). [file 13068_2015_270_MOESM1_ESM.tif]

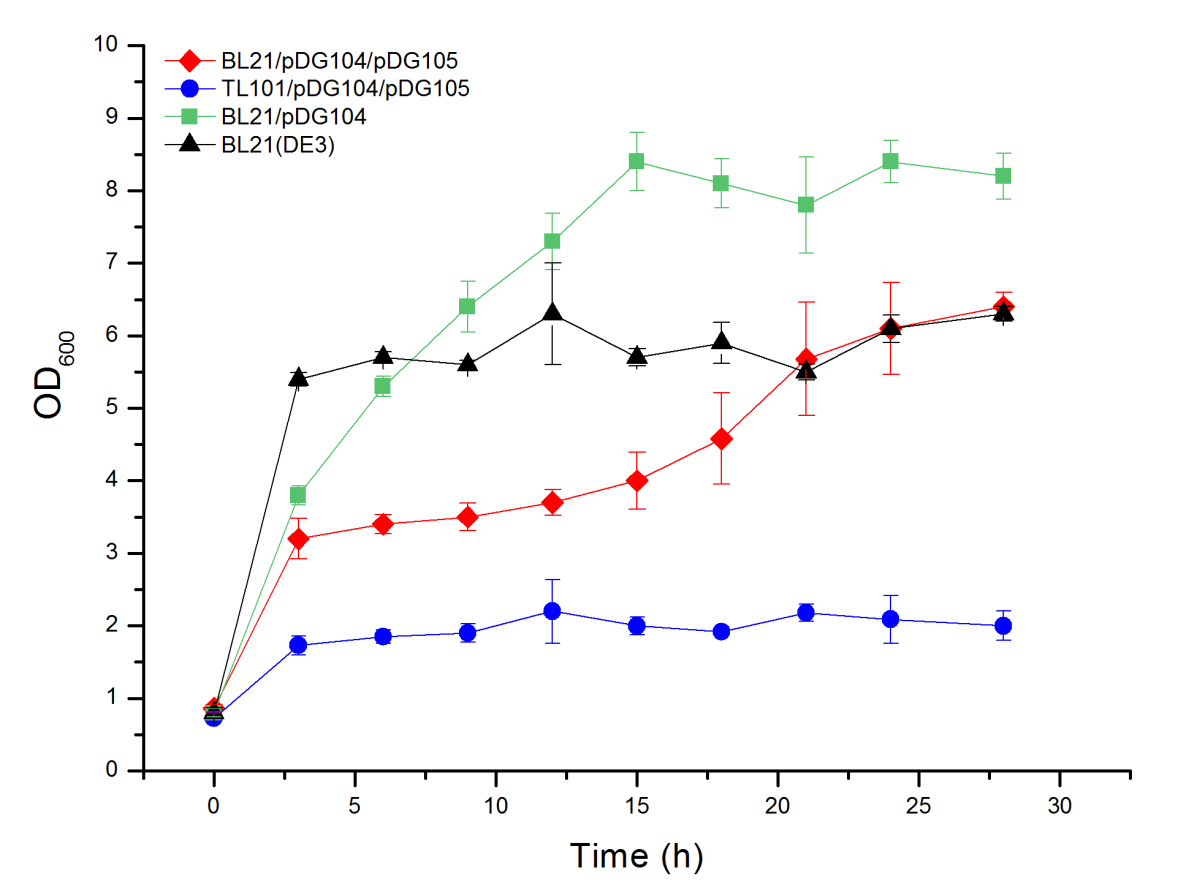

Supplement: Additional file 3: Figure S2. — OD600 curves of engineered E. coli strains in shake flasks. All experiments were performed in triplicate, and SD is indicated. [file 13068_2015_270_MOESM3_ESM.tif]

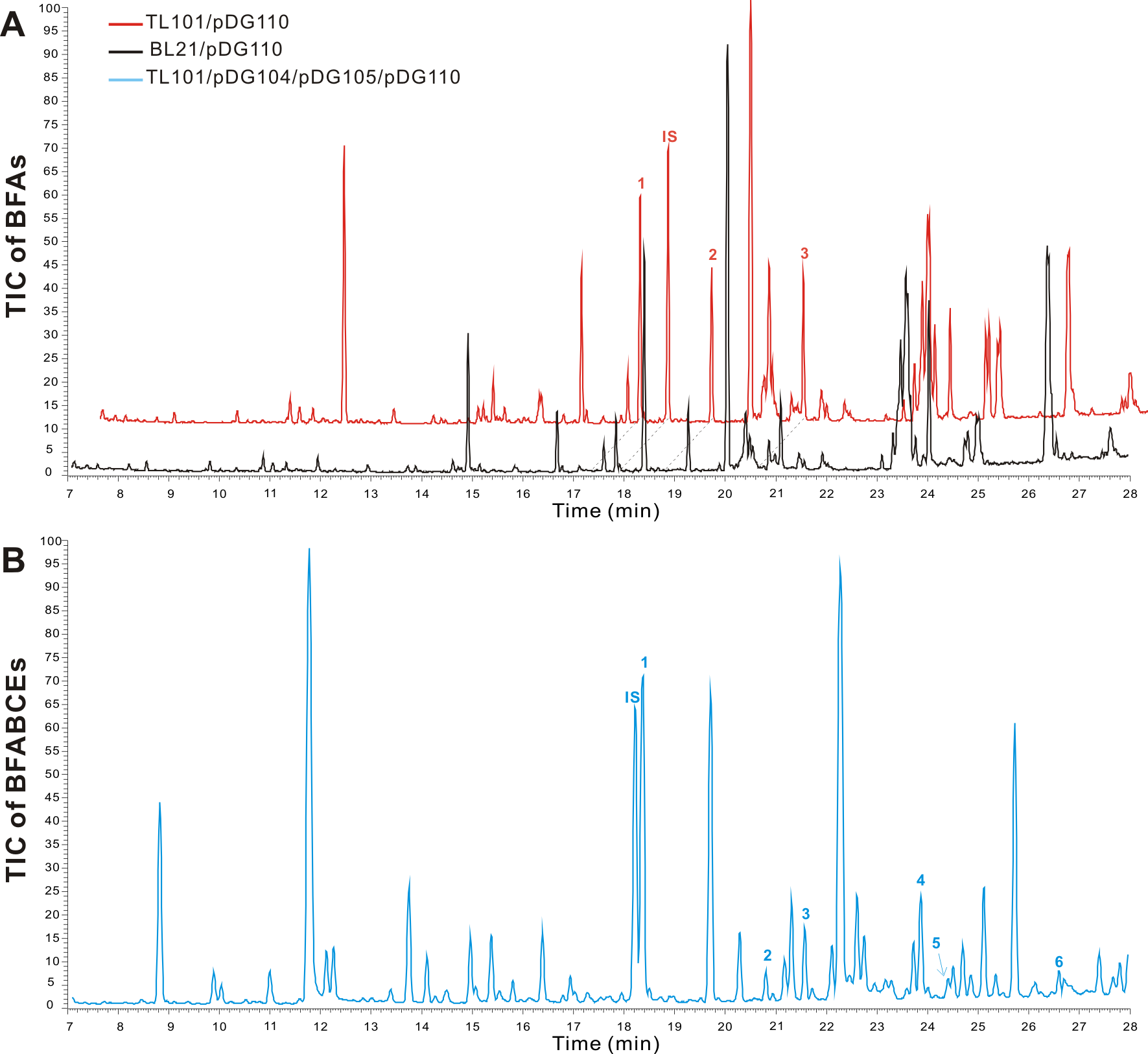

Supplement: Additional file 4: Figure S3. — GC-MS TIC of BFAs and BFABCEs in E. coli strains. All experiments were performed in triplicate. (A) GC-MS TIC of BFAs. Identified substances: (1) 12-methyltetradecanoate methyl ester; (2) 14-methylpentadecanoate methyl ester; (3) 14-methylhexadecanoate methyl ester; (IS) methyl pentadecanoic acid (internal standard). (B) GC-MS TIC of BFABCEs. Identified substances: (1) isobutyl 12-methyltridecanoate; (2) ethyl 14-methylpentadecanoate; (3) isobutyl 12-methyltetradecanoate; (4) isobutyl 14-methylpentadecanoate; (5) isoamyl 12-methyltetradecanoate; (6) isoamyl 14-methylpentadecanoate; (IS) methyl pentadecanoic acid (internal standard). [file 13068_2015_270_MOESM4_ESM.tif]

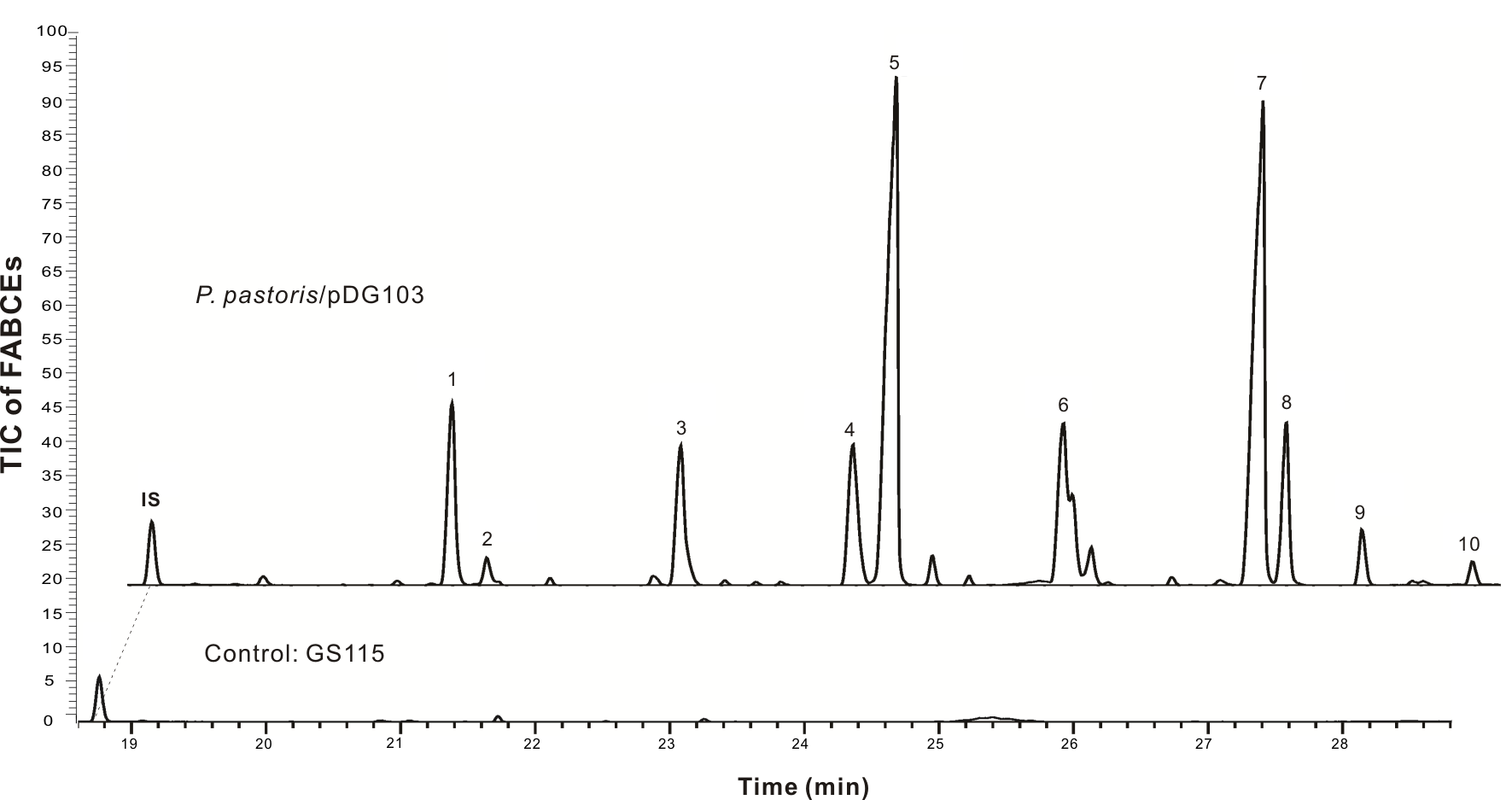

Supplement: Additional file 6: Figure S4. — GC-MS TIC of FABCEs in P. pastoris yeast. P. pastoris GS115 is the negative control. All experiments were performed in triplicate. Identified substances: (1) ethyl palmitate; (2) isoamyl myristate; (3) isobutyl palmitate; (4) ethyl stearate; (5) isoamyl palmitate; (6) isobutyl stearate; (7) isoamyl stearate; (8) isoamyl oleate; (9) isoamyl octadecadienoate; (10) isoamyl octadecatrienoate; (IS) methyl pentadecanoic acid (internal standard). [file 13068_2015_270_MOESM6_ESM.tif]
