# Supplementary material for: Metabolic engineering of microbes for branched-chain biodiesel production with low-temperature property
Source: Biotechnol Biofuels. 2015 Jun 24;8:92. doi: 10.1186/s13068-015-0270-7 (PMC4483204; doi:10.1186/s13068-015-0270-7)

Additional file 1: Synthetic fatty acid branched-chain ester (FABCE) standards. The FABCE standards were synthesized by esterification of authentic fatty acid standards and corresponding branched-chain alcohols using approaches in reference 48.

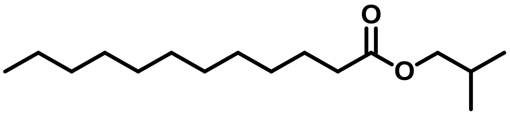

1. **Isobutyl Dodecanoate**

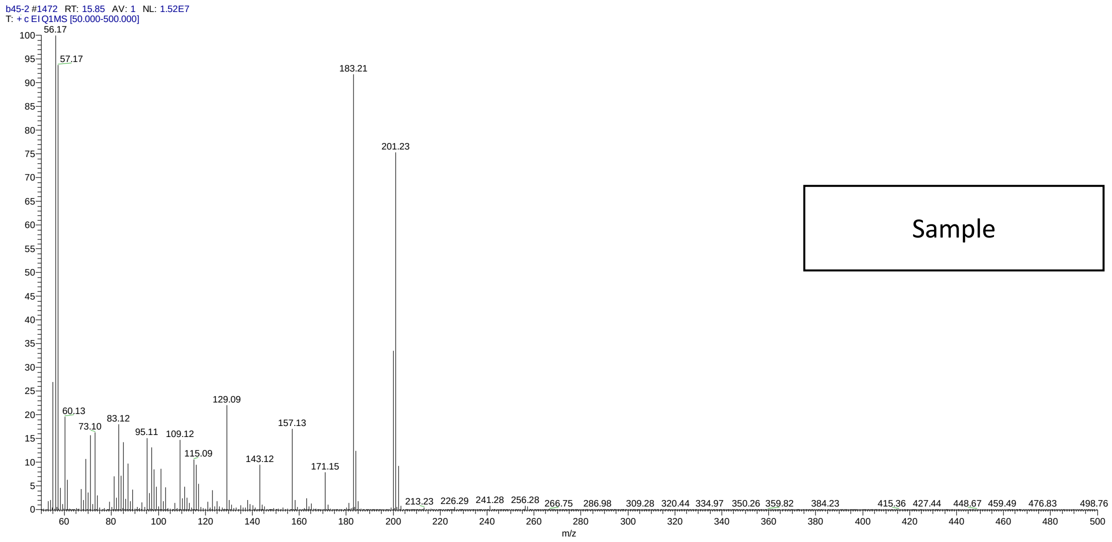

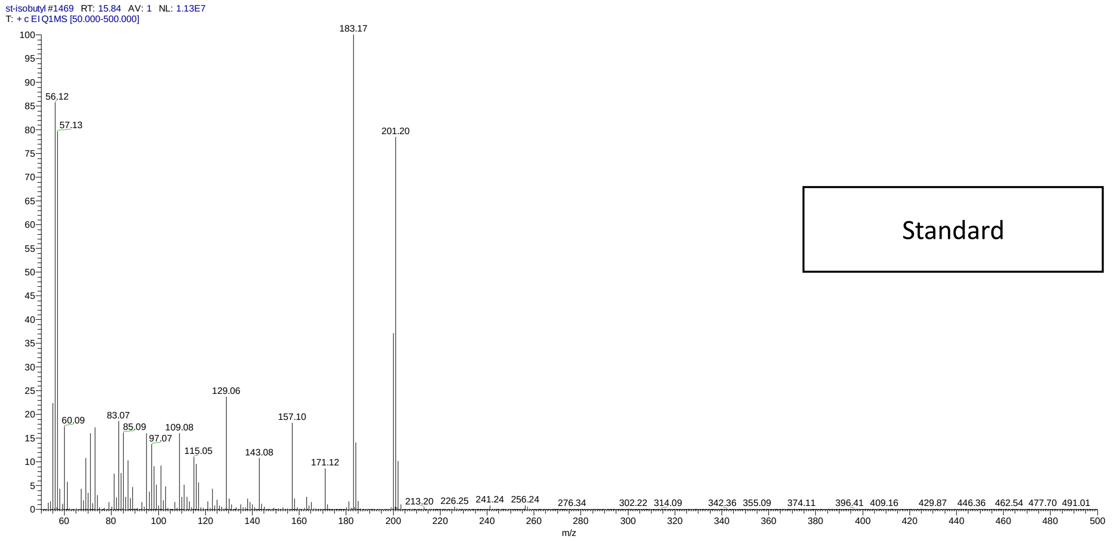

2.

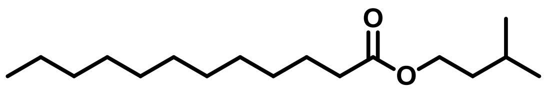

Isoamyl Dodecanoate

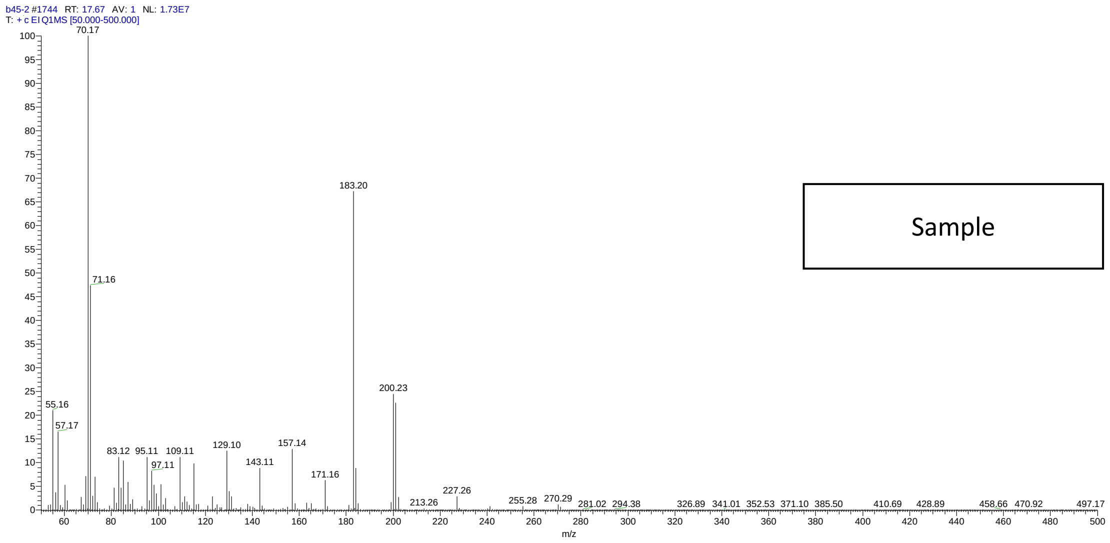

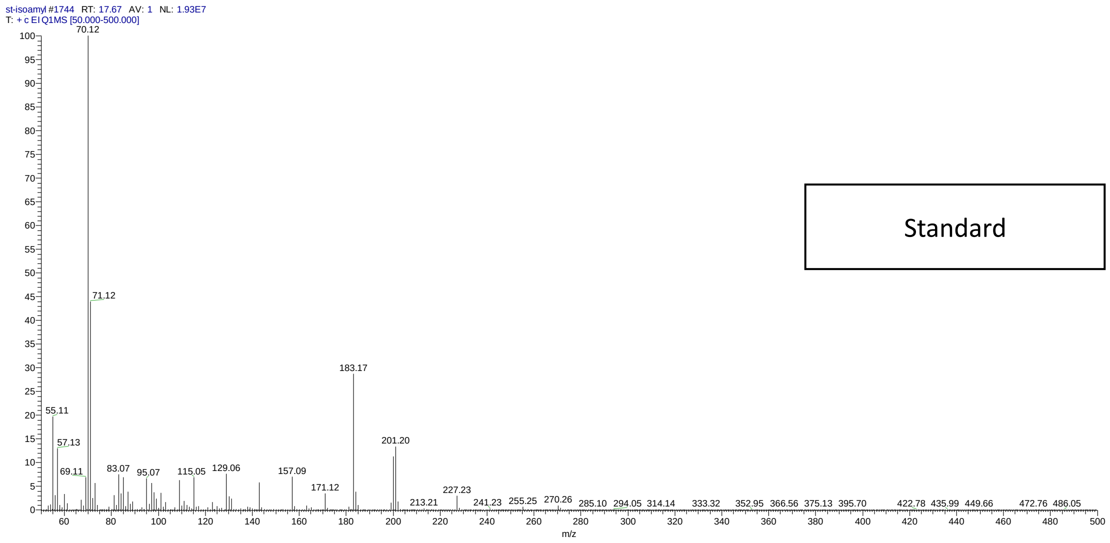

3.

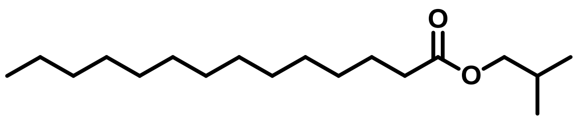

Isobutyl Myristate

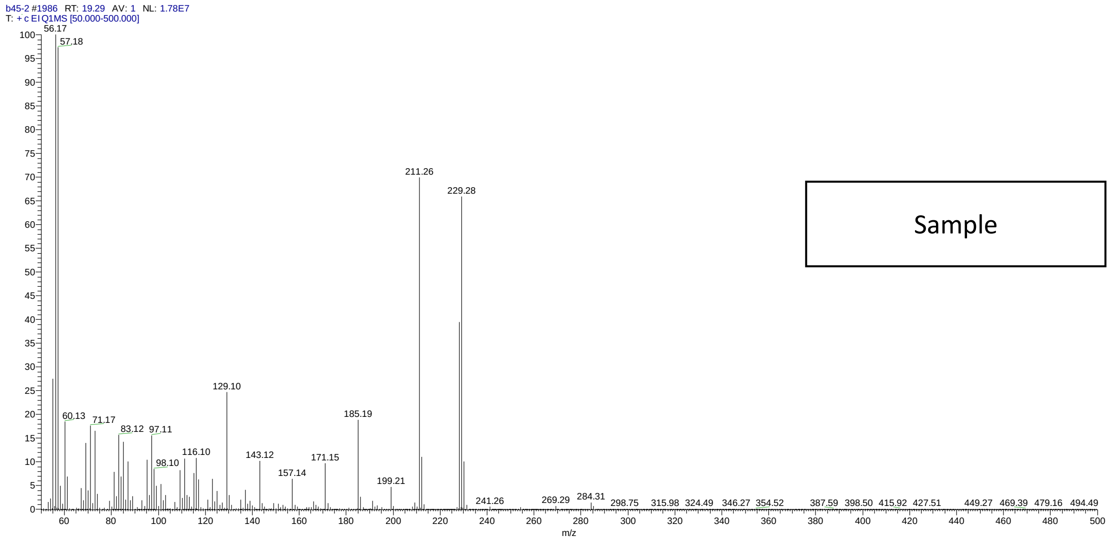

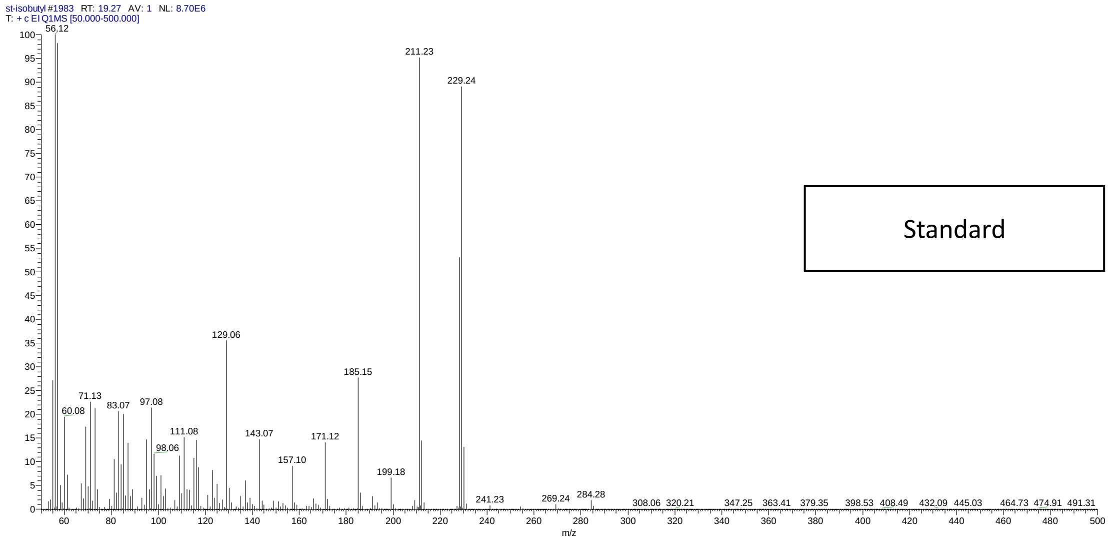

4.

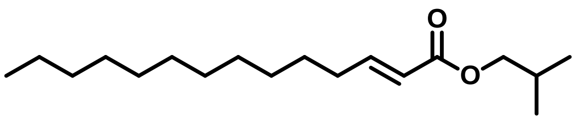

Isobutyl Tetradecenoate

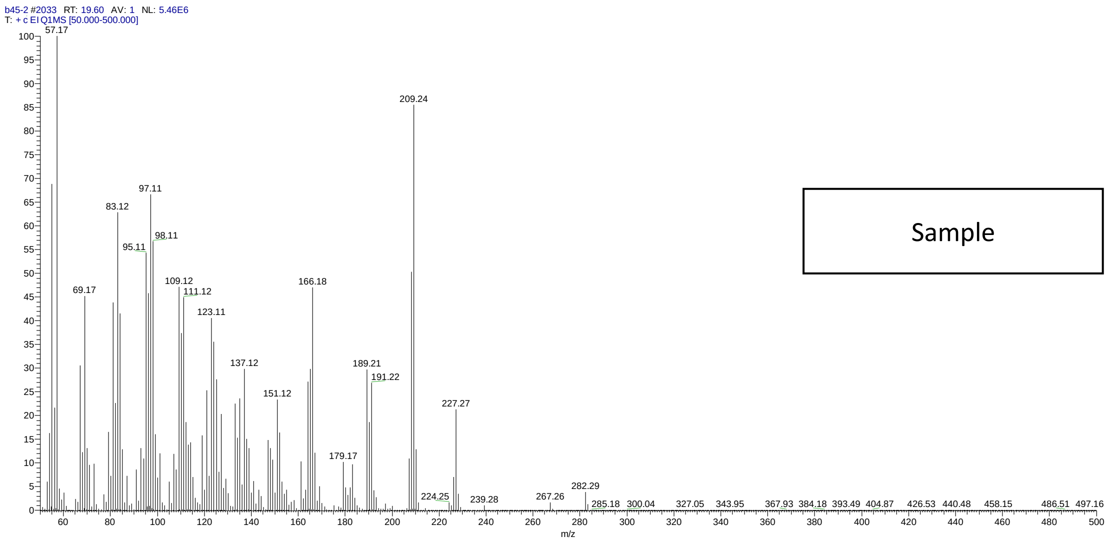

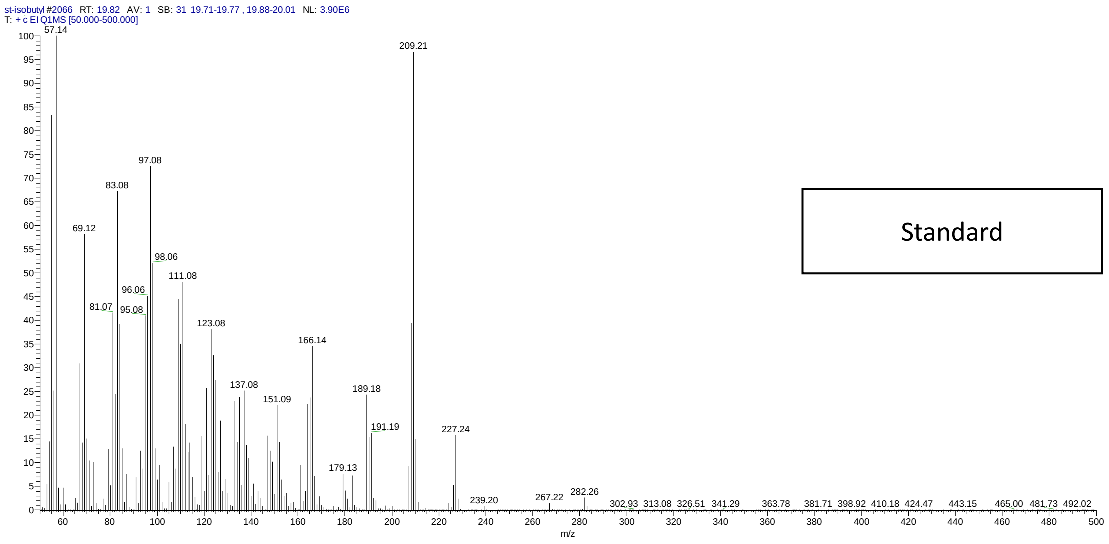

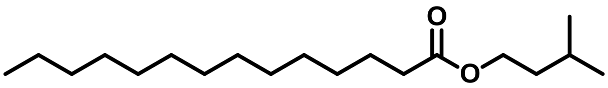

5.

Isoamyl Myristate

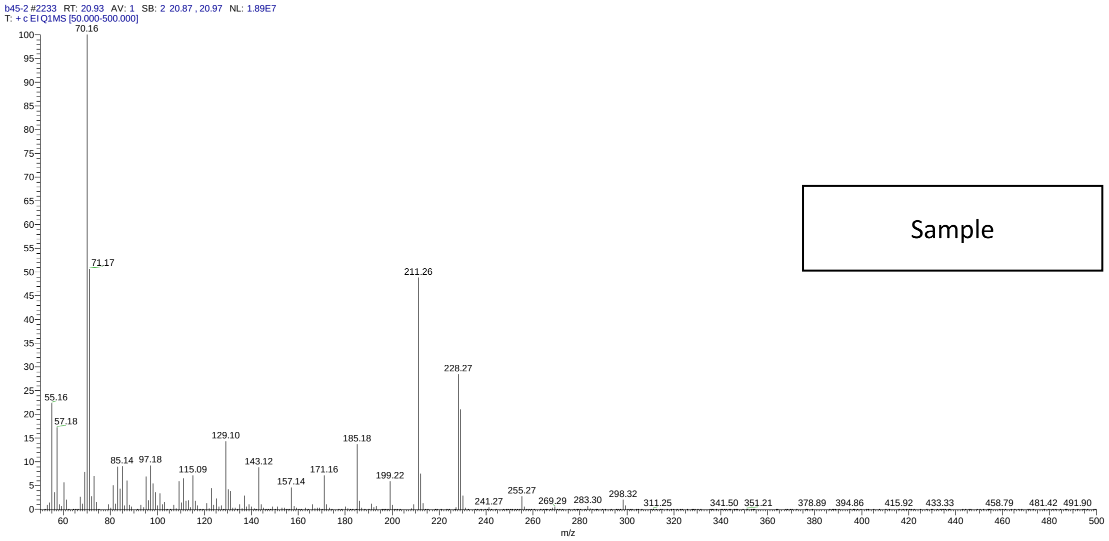

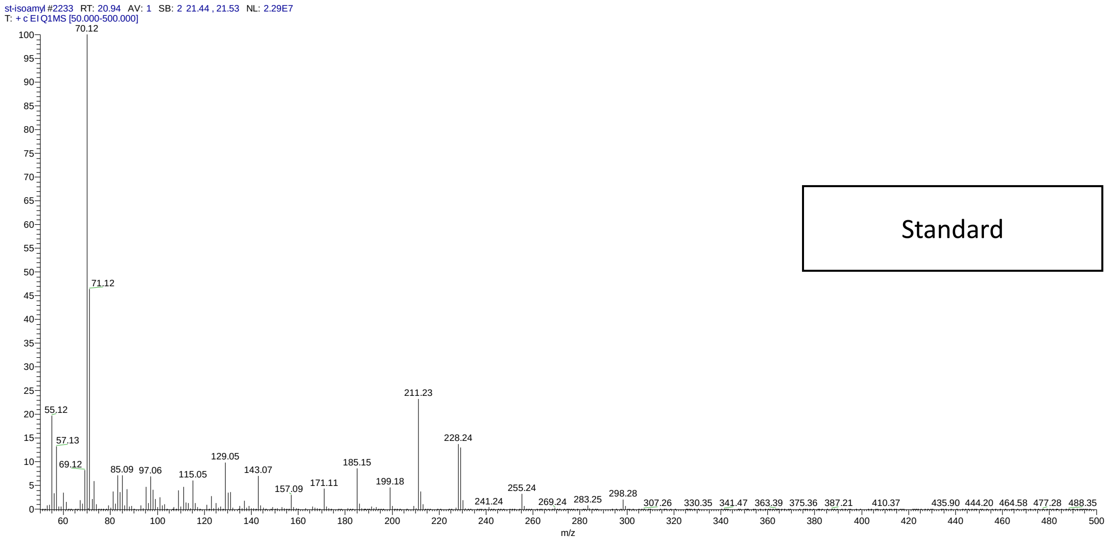

6.

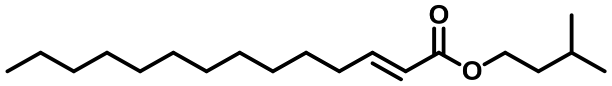

Isoamyl Tetradecenoate

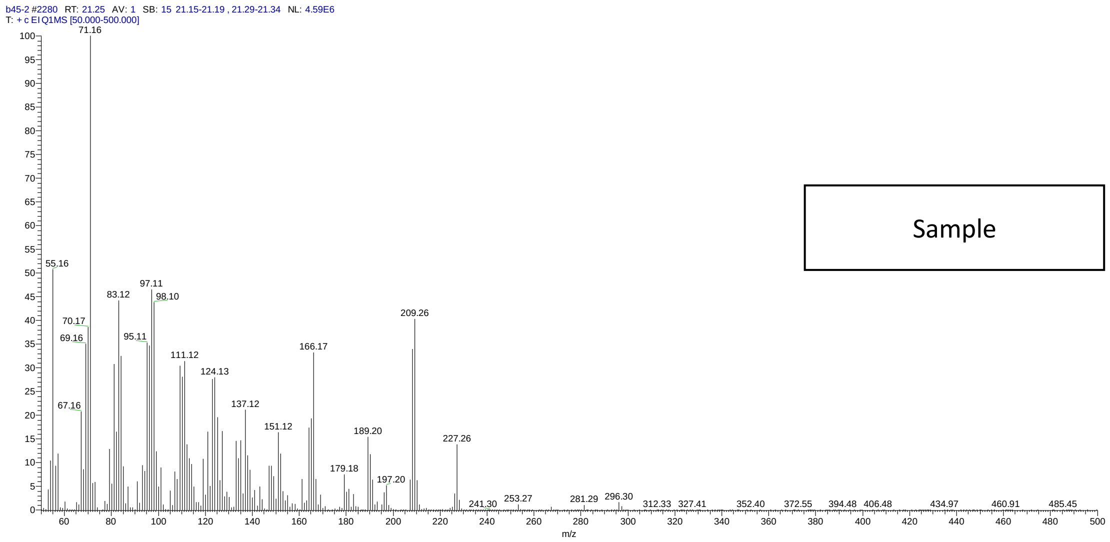

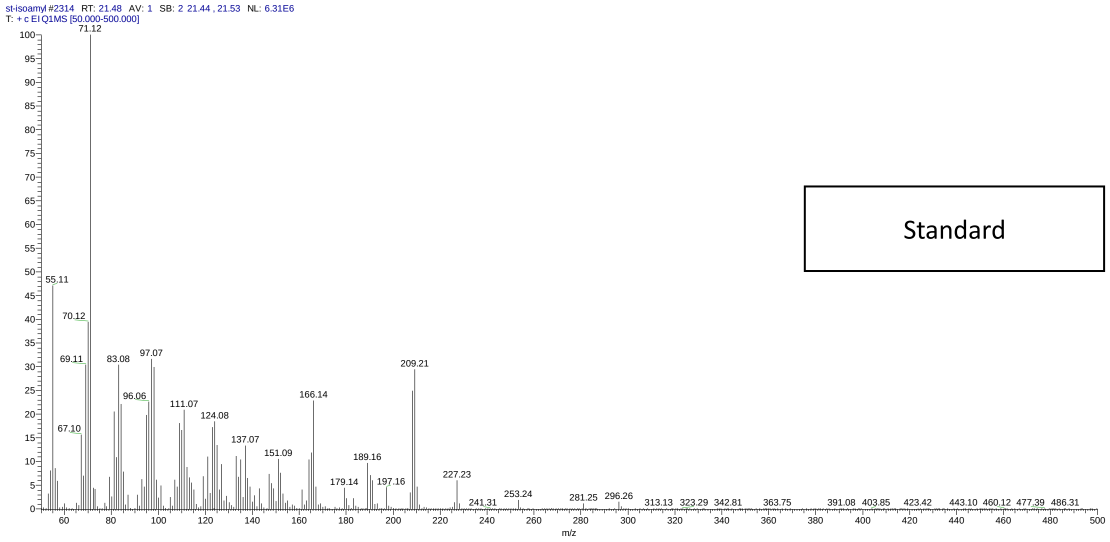

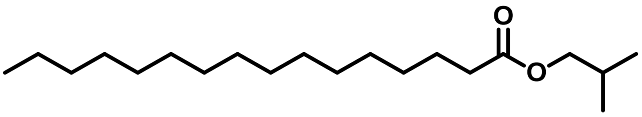

7.

Isobutyl Palmitate

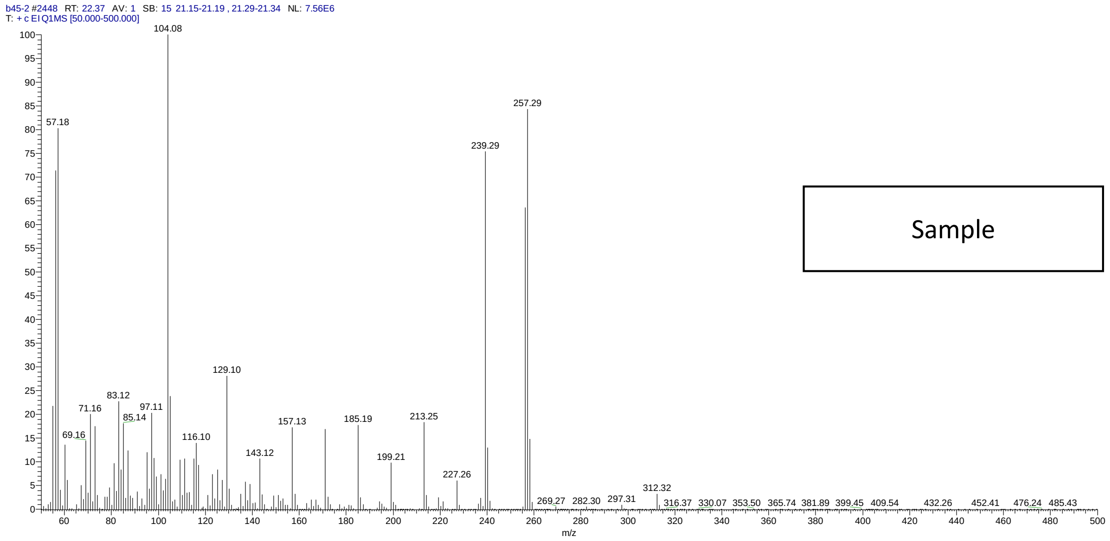

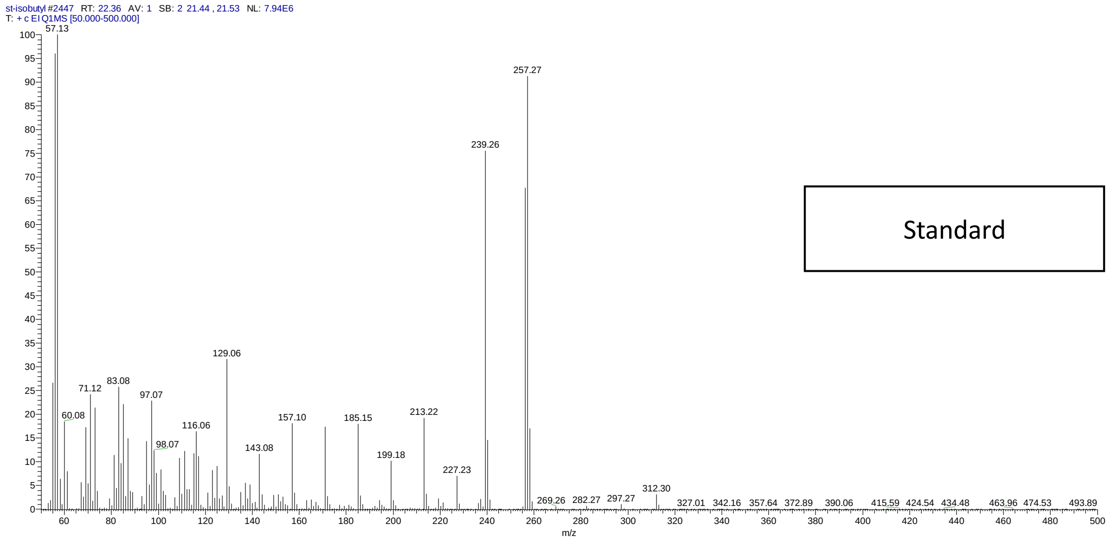

8.

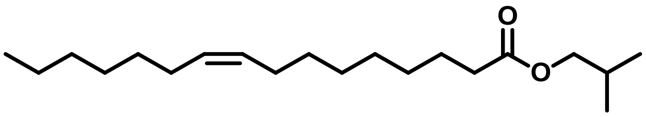

Isobutyl Palmitoleate

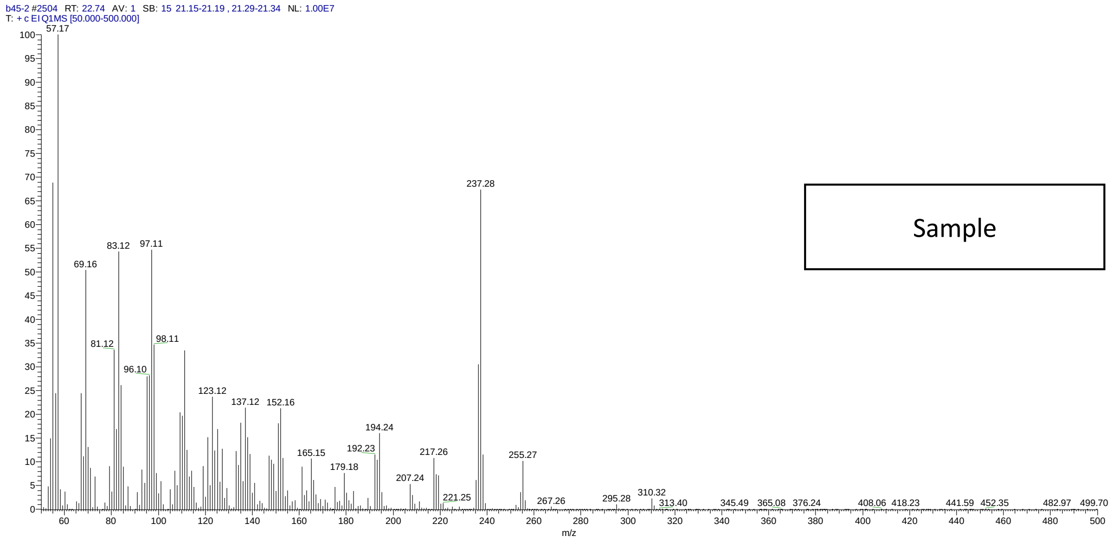

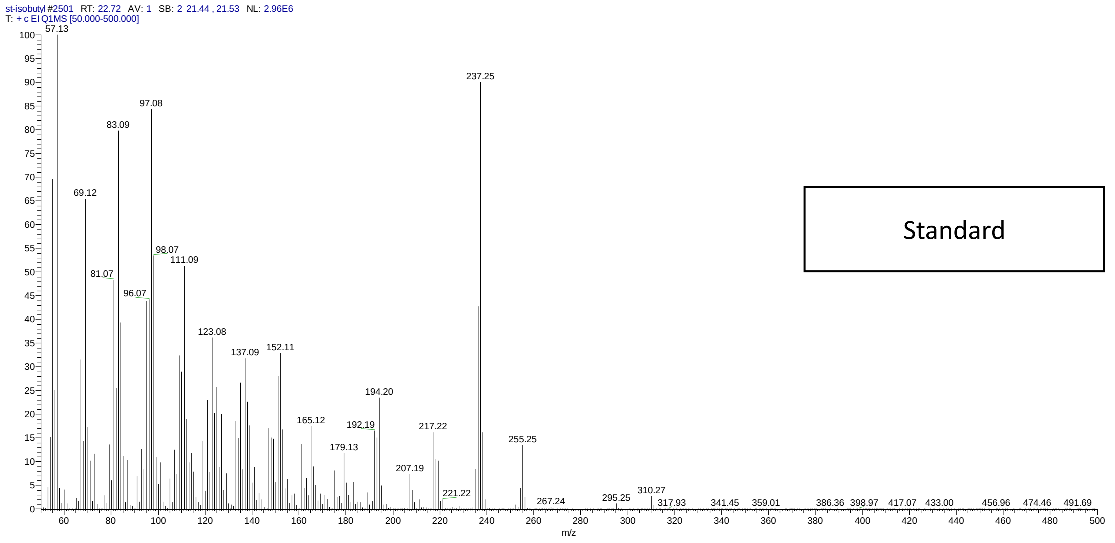

9.

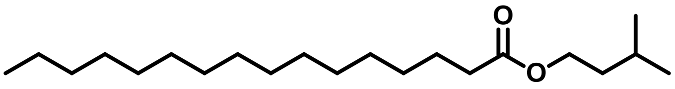

Isoamyl Palmitate

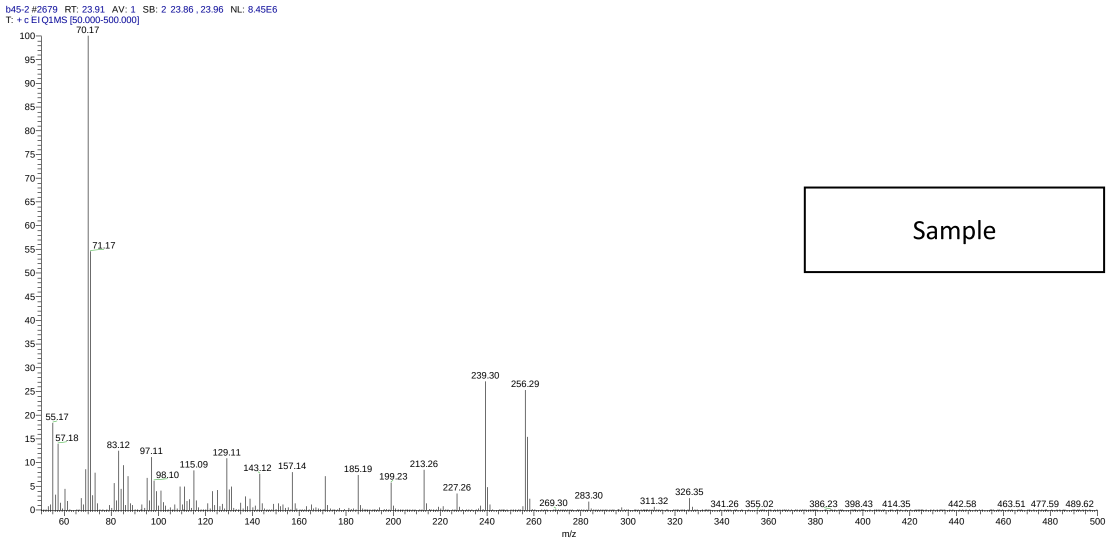

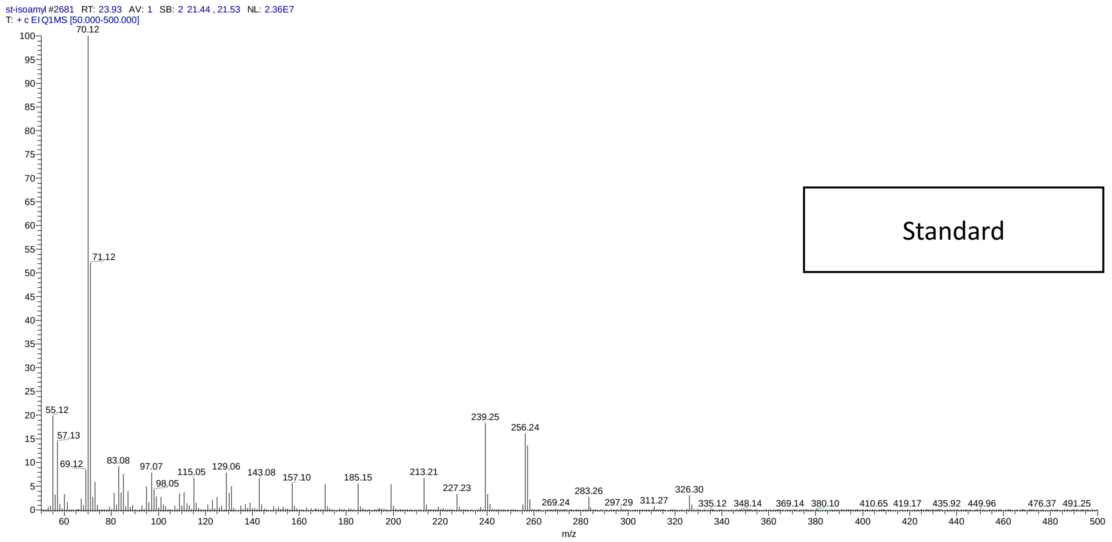

10.

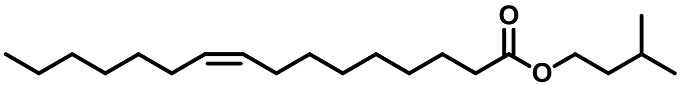

Isoamyl Palmitoleate

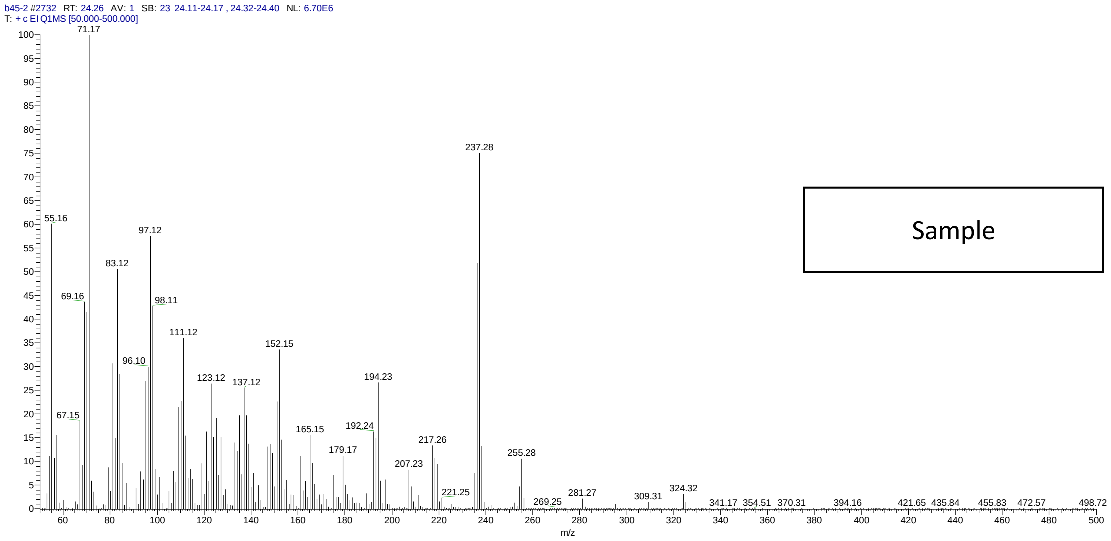

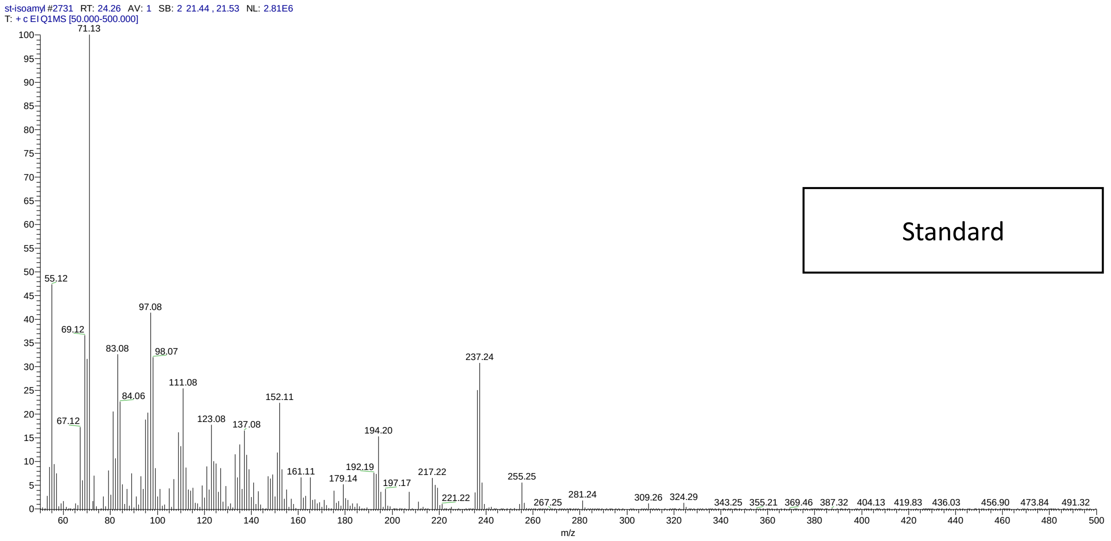

11.

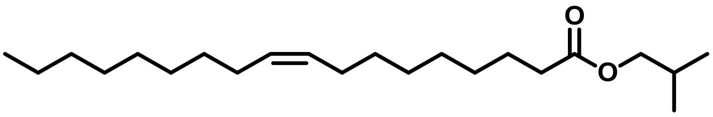

Isobutyl Oleate

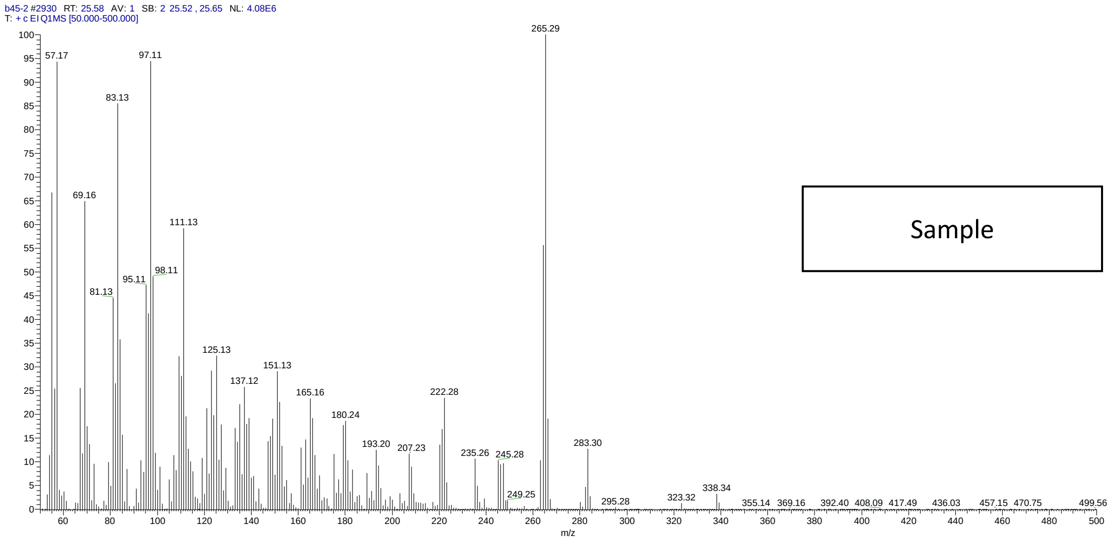

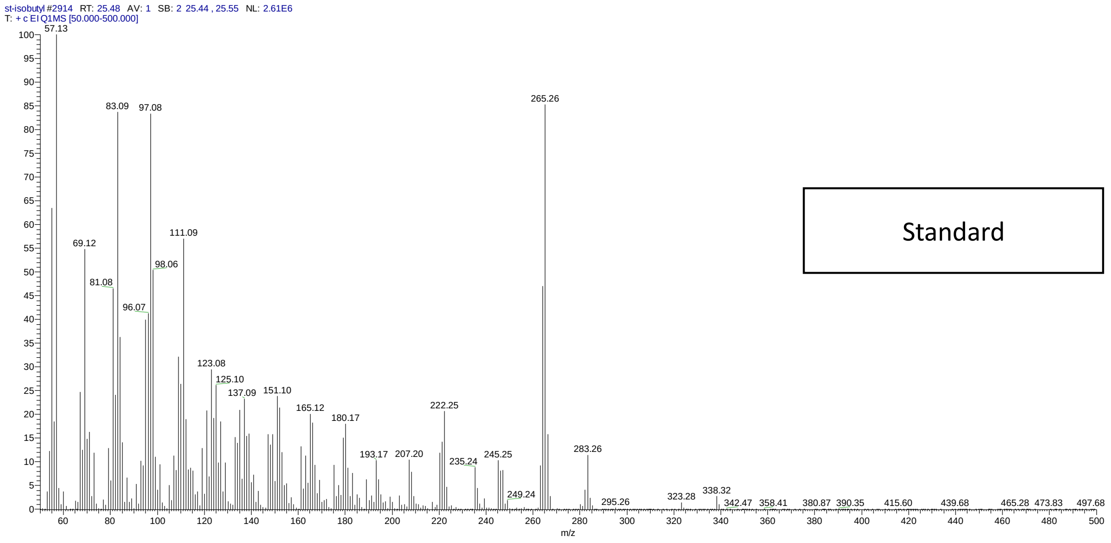

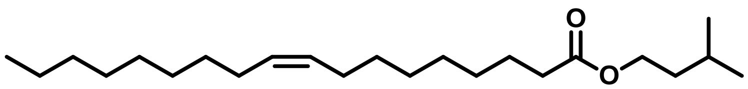

12.

Isoamyl Oleate

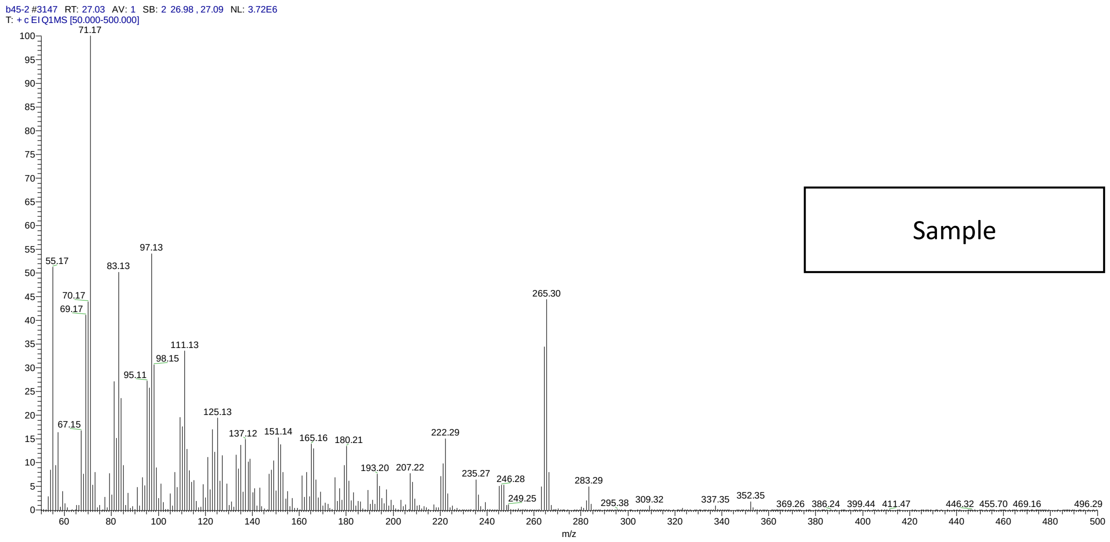

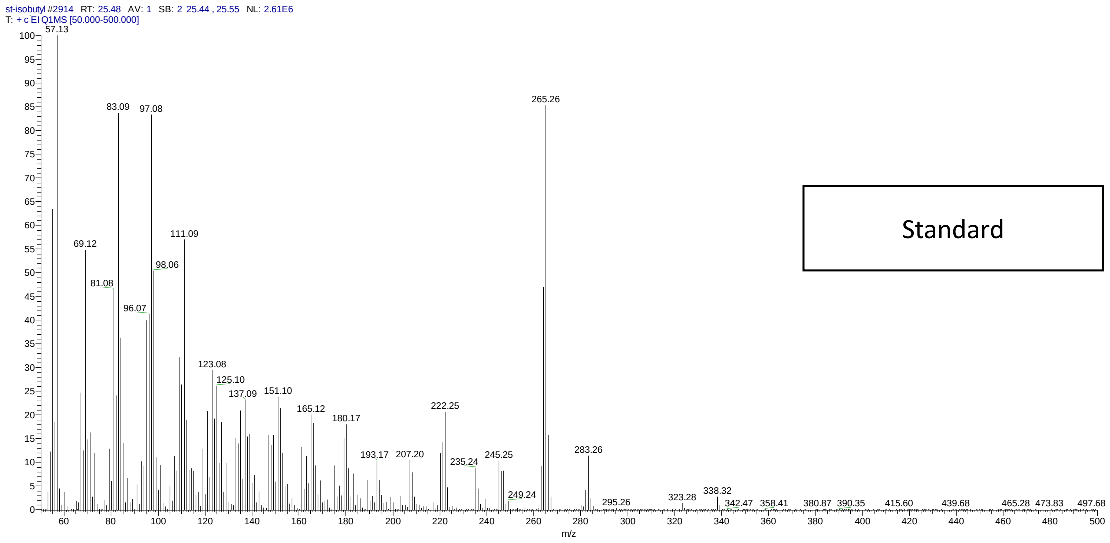

Supplement: Additional file 2: — Synthetic FABCE standards. The FABCE standards were synthesized by esterification of authentic fatty acid standards and corresponding branched-chain alcohols using approaches published in reference [48]. [file 13068_2015_270_MOESM2_ESM.pdf]
