# Supplementary material for: Metabolic engineering of microbes for branched-chain biodiesel production with low-temperature property
Source: Biotechnol Biofuels. 2015 Jun 24;8:92. doi: 10.1186/s13068-015-0270-7 (PMC4483204; doi:10.1186/s13068-015-0270-7)

Additional file 2: Synthetic branched fatty acid ester standards. The branched fatty acid ester standards were synthesized by esterification of authentic branched fatty acid standards and corresponding alcohols using approaches in reference 48.

1.

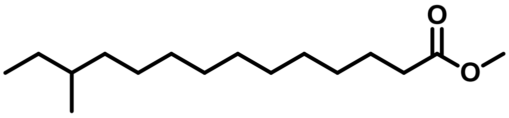

Methyl 12-Methyltetradecanoate

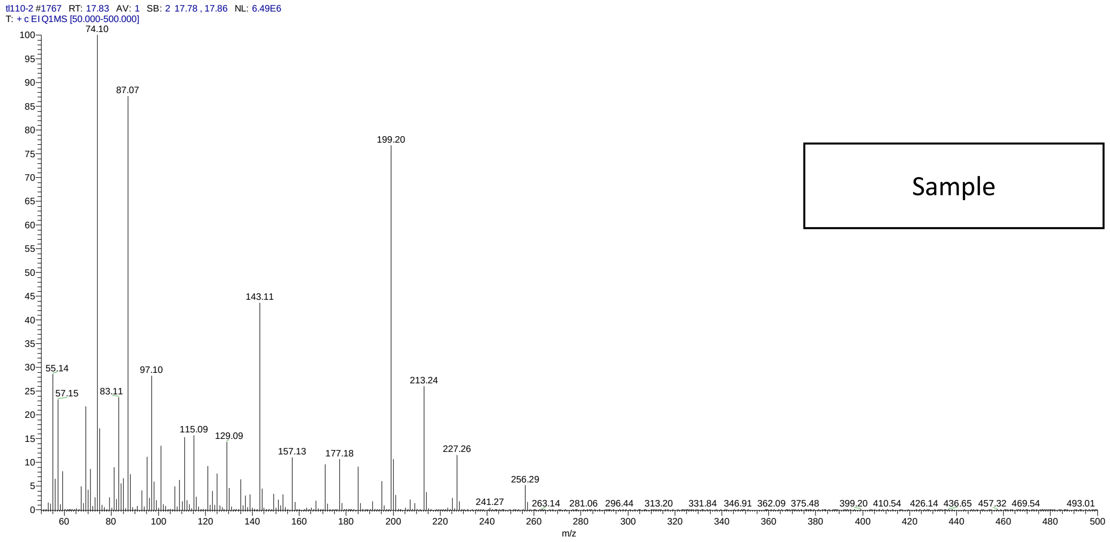

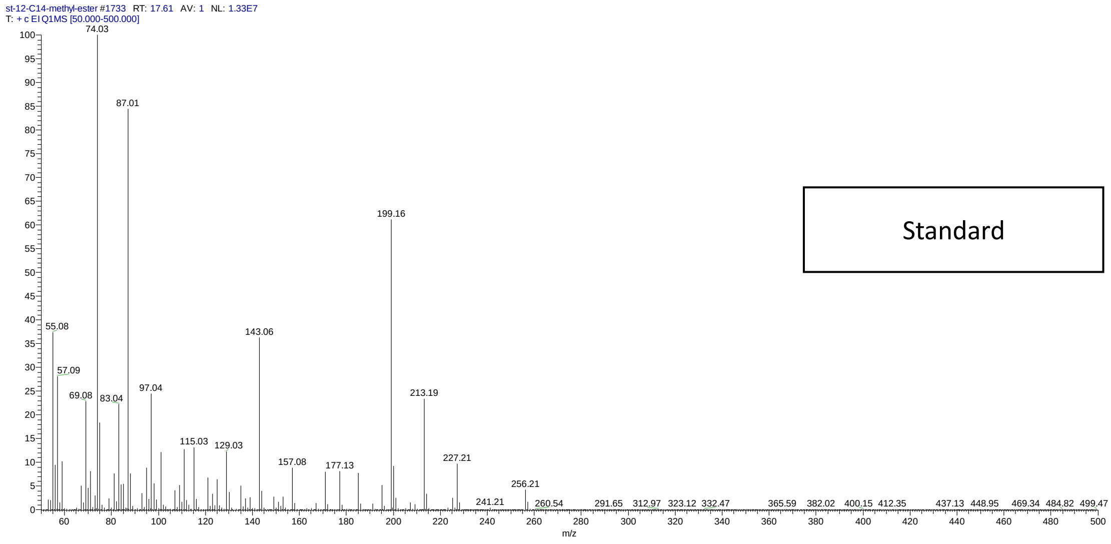

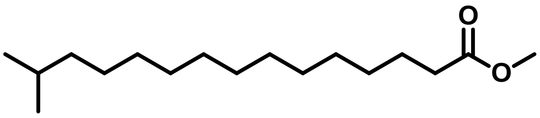

2.

methyl 14-Methylpentadecanoate

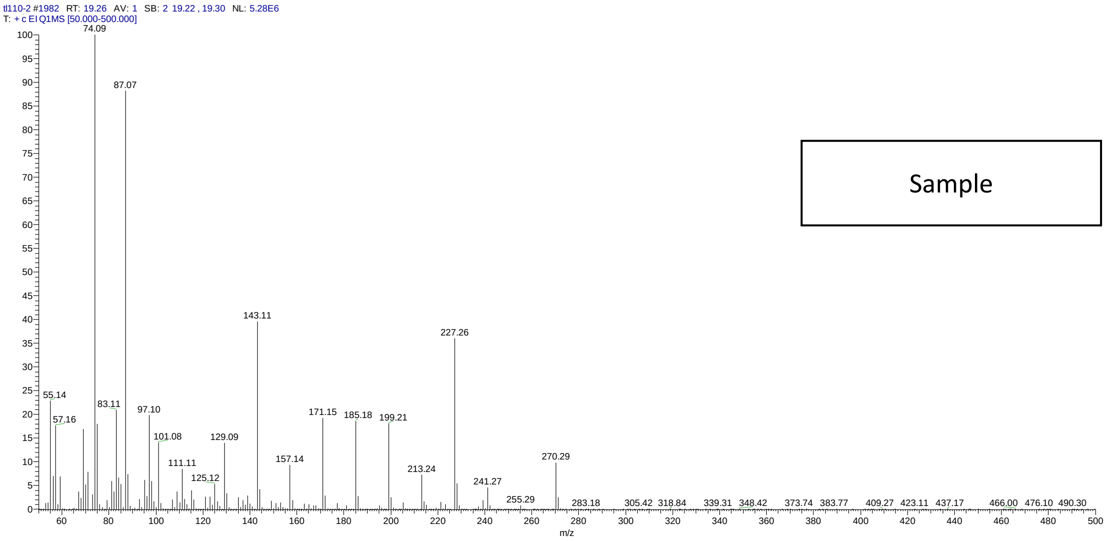

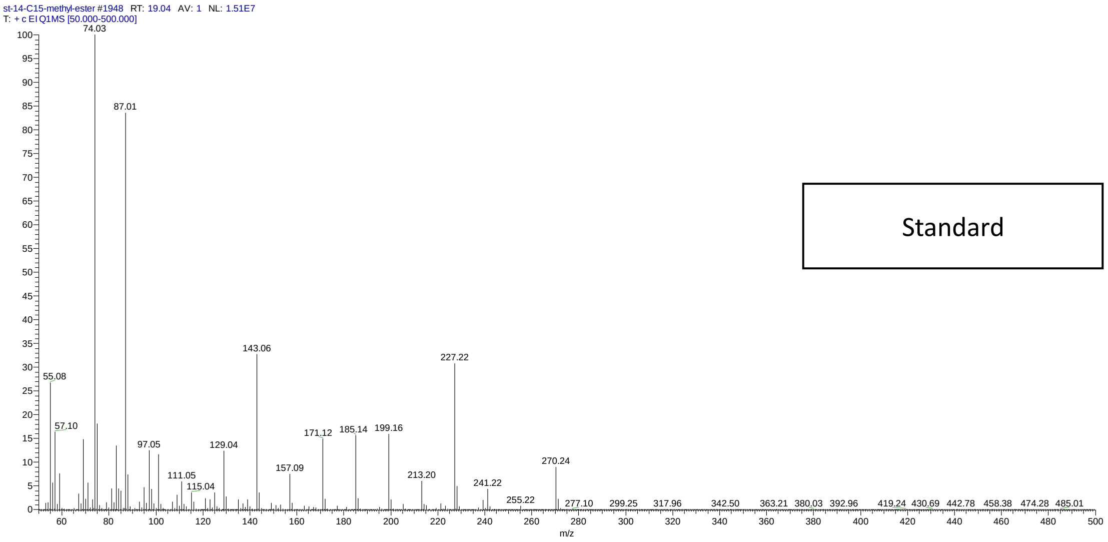

3.

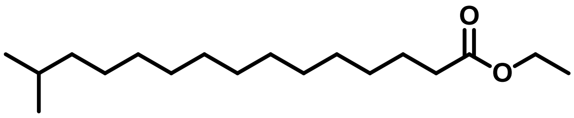

Ethyl 14-Methylpentadecanoate

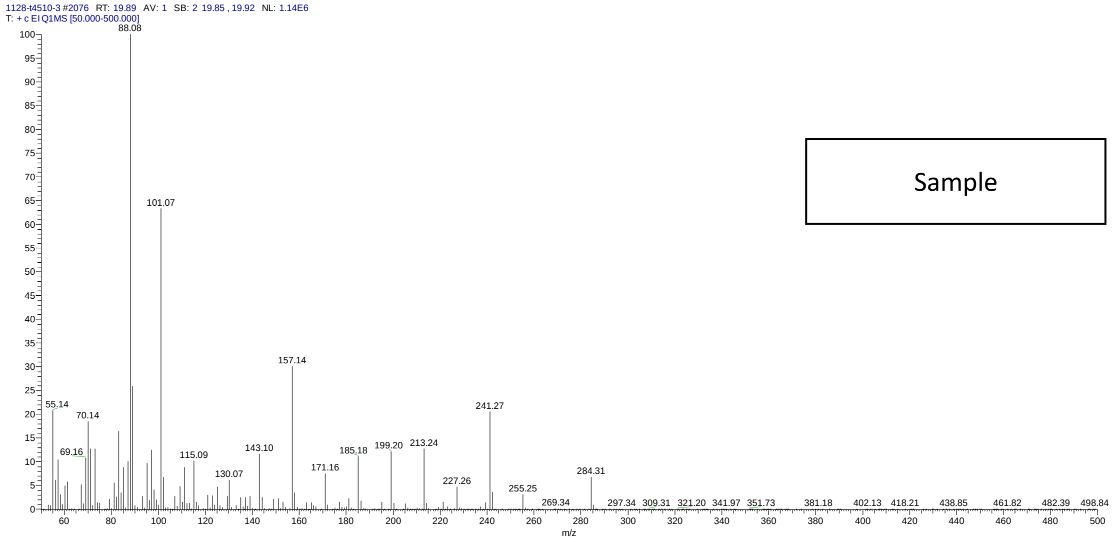

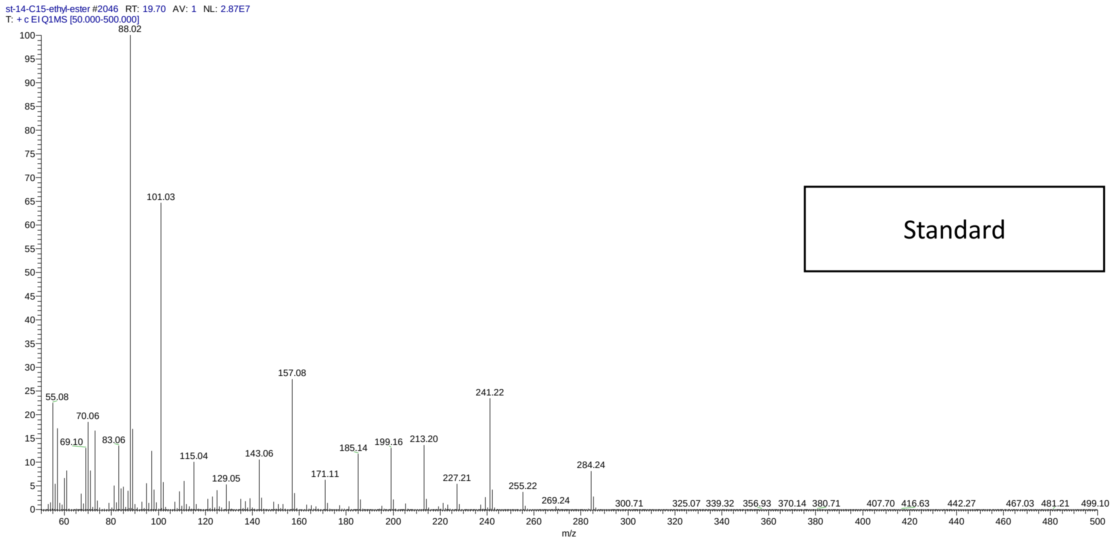

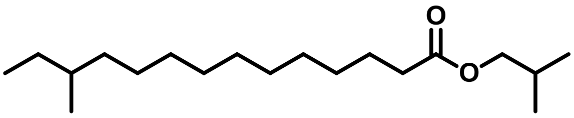

4. **Isobutyl 12-Methyltetradecanoate**

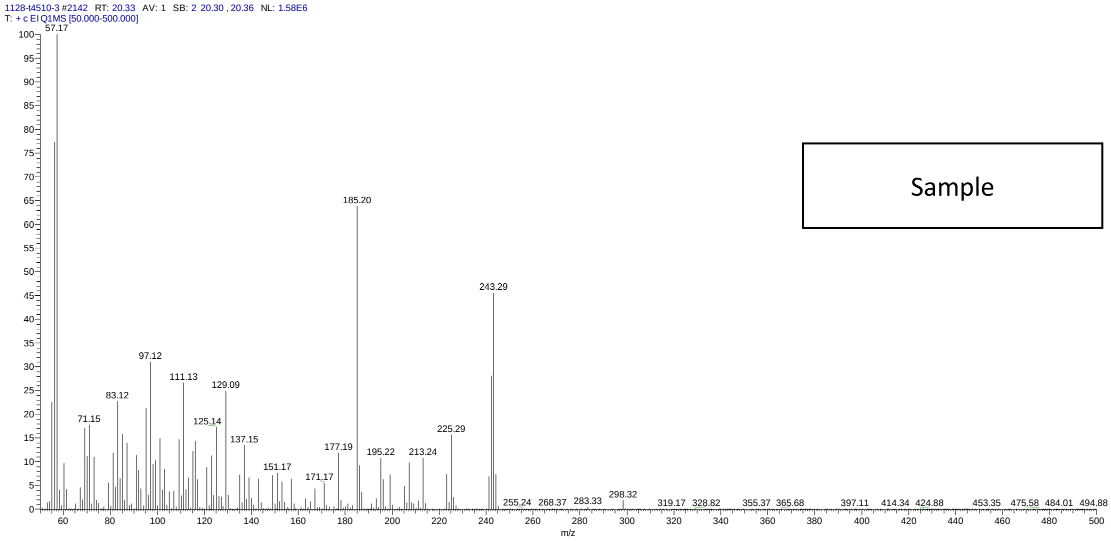

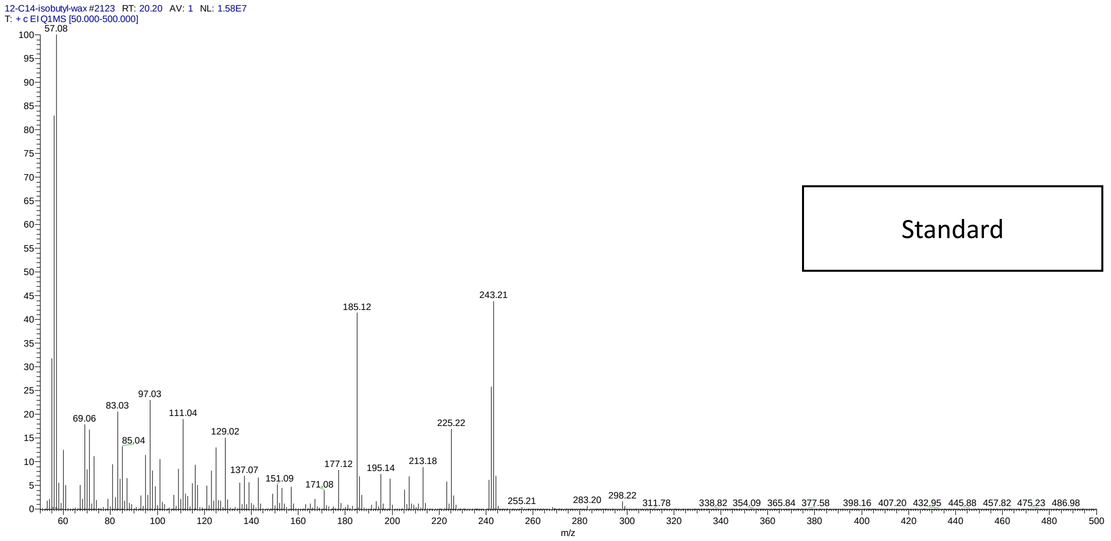

5.

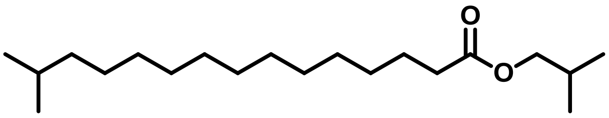

Isobutyl 14-Methylpentadecanoate

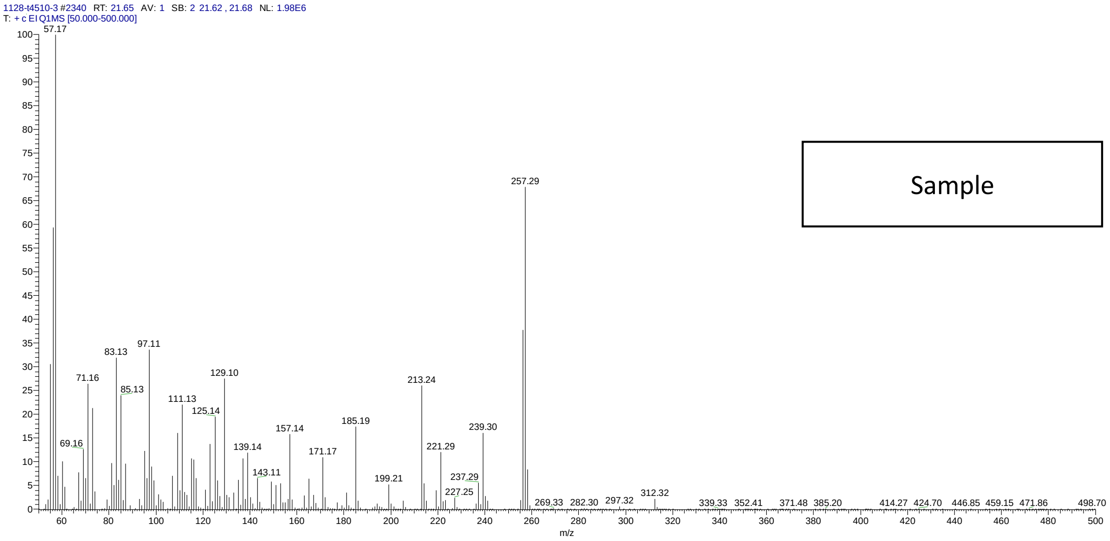

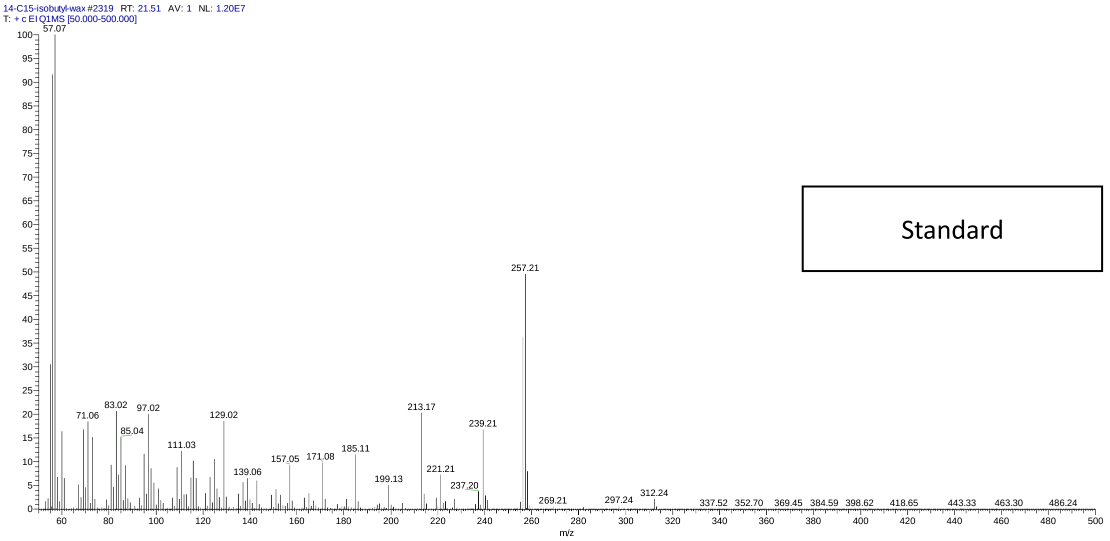

6.

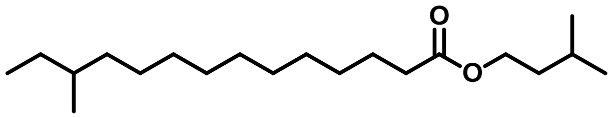

Isoamyl 12-Methyltetradecanoate

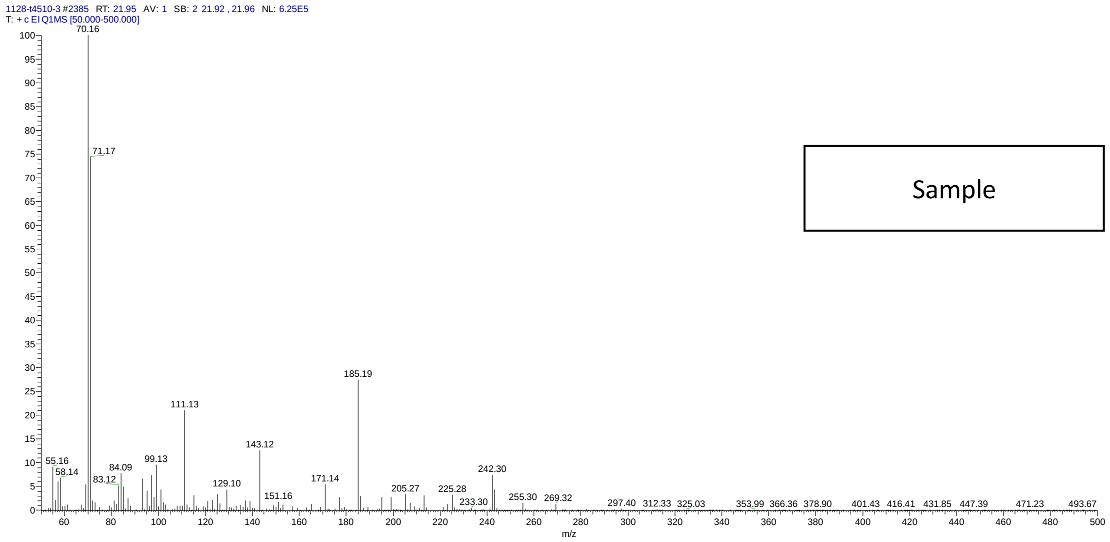

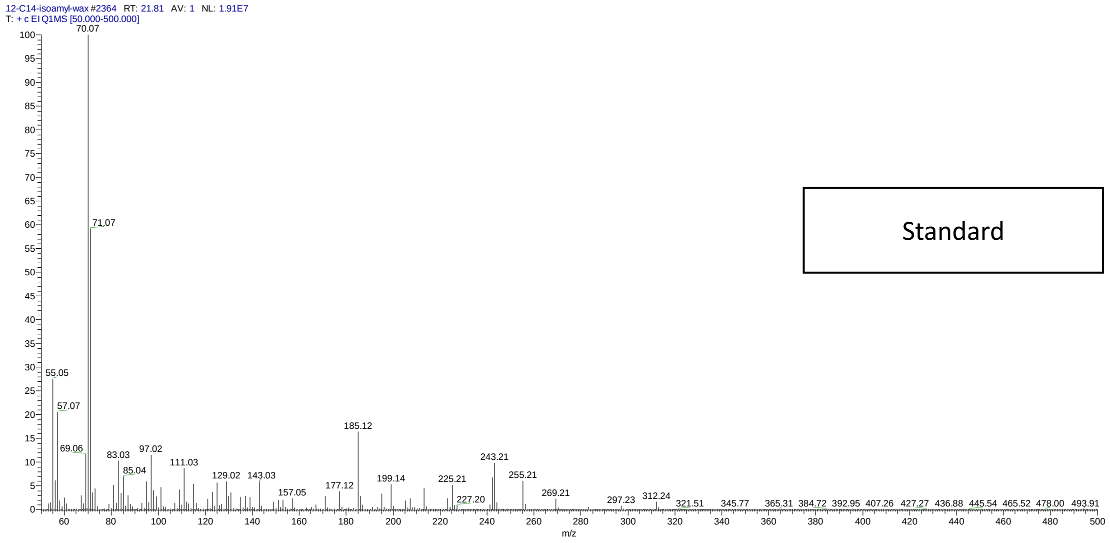

7.

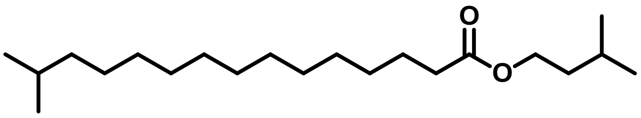

Isoamyl 14-Methylpentadecanoate

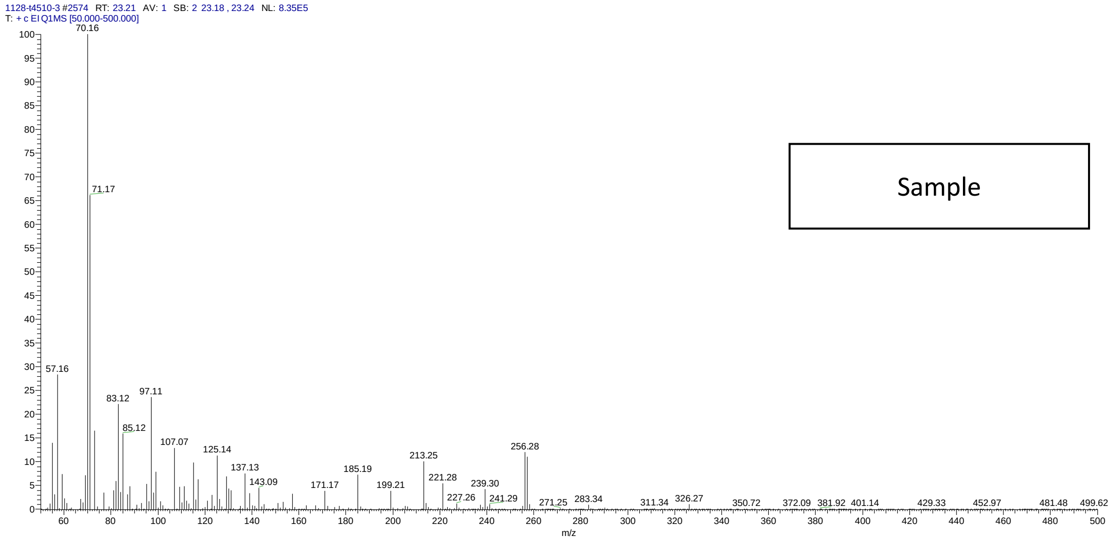

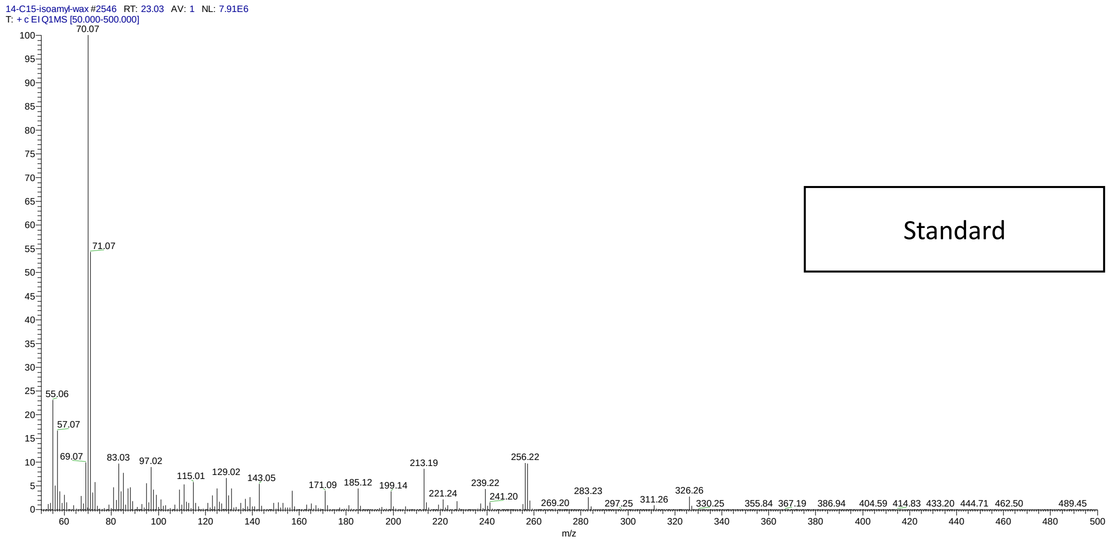

Supplement: Additional file 5: — Synthetic branched fatty acid ester standards. The branched fatty acid ester standards were synthesized by esterification of authentic branched fatty acid standards and corresponding alcohols using approaches in detailed in reference [48]. [file 13068_2015_270_MOESM5_ESM.pdf]
