# Supplementary material for: Metabolic engineering of microbes for branched-chain biodiesel production with low-temperature property
Source: Biotechnol Biofuels. 2015 Jun 24;8:92. doi: 10.1186/s13068-015-0270-7 (PMC4483204; doi:10.1186/s13068-015-0270-7)
Supplement: Additional file 7: — The sequences of synthesized ws/dgat and alsS gene. [file 13068_2015_270_MOESM7_ESM.pdf]

The sequence of *ws/dgat* gene synthesized using GenScript (Nanjing, China):

ATGAGACCATTACACCCTATTGATTTCATTTTCTTGTCTTTGGAAAAGAGAC
AACAACCAATGCATGTTGGTGGTTTGTTTTTATTCCAAATCCCAGATAATGC
ACCTGACACTTTTATTCAAGATTTGGTCAACGACATAAGAATCTCAAAGTC
CATTCCTGTTCCACCTTTCAATAACAAGTTGAACGGTTTATTTTGGGATGAA
GACGAAGAATTTGATTTGGACCATCACTTTAGACATATAGCTTTACCACACC
CTGGTAGAATCAGAGAATTGTTGATCTATATCTCCCAAGAACATAGTACTTT
GTTGGATAGAGCAAAGCCATTGTGGACATGTAACATCATCGAAGGTATCGA
GGGTAACAGATTTGCCATGTACTTCAAGATACATCACGCAATGGTAGATGGT
GTCGCCGGTATGAGATTGATCGAAAAATCTTTGTCTCACGACGTTACCGAA
AAGTCTATTGTCCCACCTTGGTGCGTTGAAGGTAAAAGAGCTAAGAGATTA
AGAGAACCAAAGACTGGTAAAATTAAGAAAATTATGTCTGGTATCAAATCA
CAATTGCAAGCAACTCCTACAGTAATTCAAGAATTGTCACAAACAGTCTTC
AAGGATATAGGTAGAAATCCAGACCATGTTTCTTCATTTCAAGCACCTTGTT
CTATTTTGAACCAAAGAGTATCCAGTTCTAGAAGATTTGCTGCACAATCCTT
CGATTTGGACAGATTCAGAAACATCGCCAAGAGTTTGAACGTTACCATAAA
CGATGTTGTATTAGCTGTATGCTCTGGTGCCTTGAGAGCTTATTTGATGTCAC
ATAATTCCTTGCCTAGTAAGCCTTTAATCGCTATGGTACCAGCATCAATTAGA
AATGATGACTCTGATGTCTCAAACAGAATCACAATGATCTTGGCAAATTTG
GCCACCCACAAAGATGACCCTTTGCAAAGATTAGAAATCATCAGAAGATCC
GTTCAAAACAGTAAGCAAAGATTCAAGAGAATGACTTCTGATCAAATATTG
AACTACTCAGCTGTCGTTTACGGTCCAGCAGGTTTAAACATAATCTCTGGTA
TGATGCCTAAGAGACAAGCCTTTAATTTGGTTATTTCAAACGTACCAGGTCC
TAGAGAACCATTATATTGGAATGGTGCCAAGTTGGATGCTTTATACCCTGCA
TCCATAGTTTTGGACGGTCAAGCTTTAAACATCACCATGACTTCTTACTTGG
ATAAGTTAGAAGTTGGTTTGATTGCCTGTAGAAATGCTTTACCAAGAATGCA
AAACTTGTTAACACATTTGGAAGAAGAAATCCAATTGTTTGAAGGTGTTAT
CGCCAAGCAAGAAGACATTAAGACTGCCAACTAA

The sequence of *alsS* gene synthesized using GenScript (Nanjing, China):

ATGACAAAGGCCACCAAGGAGCAGAAGAGCCTGGTGAAGAACCGCGGTG
CCGAACTGGTTGTTGACTGCCTGGTGGAACAGGGCGTGACCCACGTGTTC
GGCATTCCGGGCGCAAAGATCGATGCCGTGTTCGACGCCCTGCAGGACAA
GGGCCCGGAGATTATTGTTGCCCGCCATGAACAGAATGCCGCCTTCATGGC
ACAAGCCGTTGGCCGCTTAACAGGCAAGCCGGGCGTGGTTTTAGTTACCA
GCGGTCCGGGTGCAAGCAATTTAGCAACAGGCCTGCTGACAGCCAACACC
GAAGGTGACCCTGTGGTGGCCCTGGCAGGCAATGTGATCCGCGCAGATCG
CCTGAAACGCACCCACCAAAGTCTGGATAACGCAGCCCTGTTCCAGCCGAT
TACCAAGTATAGCGTGGAGGTTCAGGACGTGAAGAACATCCCTGAGGCCG
TGACCAACGCCTTTCGCATCGCAAGTGCAGGCCAGGCAGGCGCCGCCTTT
GTGAGCTTCCCGCAGGATGTGGTGAACGAAGTGACCAACACCAAGAACGT
GCGTGCAGTTGCCGCACCTAAGCTGGGCCCTGCCGCAGACGATGCAATTAG
TGCCGCCATCGCAAAGATCCAGACCGCCAAGCTGCCTGTGGTTCTGGTGG
GCATGAAAGGTGGCCGTCCTGAGGCAATCAAGGCAGTGCGCAAGCTGCTG
AAAAAGGTGCAGCTGCCTTTCGTGGAGACCTACCAGGCCGCCGGTACACT
GAGCCGCGATCTGGAGGACCAGTACTTCGGTCGCATCGGCTTATTCCGCAA
CCAGCCGGGCGACTTACTGCTGGAGCAAGCAGATGTGGTGCTGACCATCG
GCTACGACCCGATCGAGTACGACCCTAAGTTCTGGAACATCAACGGTGACC
GCACCATCATCCACCTGGACGAGATCATCGCAGACATTGATCACGCATACC
AGCCGGATCTGGAACTGATTGGTGATATCCCGAGCACCATCAACCACATCG
AACACGACGCCGTTAAGGTTGAGTTCGCAGAGCGCGAGCAGAAAATACTG
AGTGACCTGAAGCAATACATGCATGAGGGCGAGCAAGTTCCGGCCGACTG
GAAGAGTGATCGTGCCCATCCGCTGGAGATCGTGAAAGAACTGCGCAACG
CCGTGGACGACCACGTGACAGTGACCTGCGACATTGGCAGTCATGCCATCT
GGATGAGTCGCTACTTCCGTAGCTACGAGCCGCTGACCCTGATGATCAGCA
ATGGCATGCAGACCCTGGGCGTGGCATTACCGTGGGCCATCGGTGCAAGCC
TGGTGAAACCTGGCGAAAAGGTGGTGAGCGTGAGCGGCGATGGTGGTTTT
CTGTTCAGCGCAATGGAACTGGAAACCGCCGTGCGTTTAAAGGCCCCGATC
GTTCACATCGTGTGGAACGACAGTACCTACGACATGGTGGCATTCCAGCAG
CTGAAGAAGTATAACCGCACCAGTGCCGTTGACTTTGGCAACATCGACATT

GTGAAGTACGCCGAGAGTTTCGGTGCCACAGGCTTACGCGTGGAAAGCCC

GGATCAACTGGCCGATGTTCTGCGTCAGGGCATGAACGCCGAGGGCCCTG

TGATTATCGACGTGCCGGTGGACTACAGCGATAACATCAATCTGGCAAGCG

ACAAGCTGCCTAAGGAGTTCGGCGAGCTGATGAAGACCAAAGCACTGTAA
